# The role of selection pressure in shaping zoxamide resistance in *Plasmopara viticola* populations

**DOI:** 10.1002/ps.8890

**Published:** 2025-05-12

**Authors:** Mattia Peracchi, Giuliana Maddalena, Beatrice Lecchi, Federico Massi, Silvia Laura Toffolatti

**Affiliations:** ^1^ University of Milan Department of Agricultural and Environmental Sciences – DiSAA Milan Italy

**Keywords:** fungicide sensitivity, target‐site mutation, downy mildew control, fungicide resistance management, oomycete, anti‐resistance strategy

## Abstract

**BACKGROUND:**

Zoxamide, a *β‐tubulin* inhibitor, is widely used in vineyards to control downy mildew caused by the high‐risk pathogen *Plasmopara viticola*. This study aimed to investigate the selection of zoxamide resistance and its characterization, providing practical insights for resistance management, through a twofold approach: a quantitative assessment of selection pressure effects on oospore populations and the molecular characterization of resistance‐associated mutations in *P. viticola* strains.

**RESULTS:**

A total of 126 populations sampled from 57 vineyards mainly located in North‐eastern Italy, were analyzed over a 6‐year period (2017–2022). Based on toxicological parameters, 90% of the samples were fully sensitive to zoxamide (EC_50_ < 0.2 mg/L; EC_95_ and MIC<10 mg/L). Resistant individuals, able to germinate at 100 mg/L zoxamide, were detected in low frequency (<12%) within 13 samples. Only two samples showed a high frequency of resistant individuals (24–33%). Resistance was primarily found in vineyards treated more than four times per season with zoxamide. Partial sequencing of *β‐tubulin* gene revealed different polymorphisms at codon 239 associated with resistant isolates: the known C239S/G mutations, with the SG genotype being predominant, and a potential novel C239T mutation, not previously reported.

**CONCLUSION:**

This study highlighted a low risk of resistance under moderate fungicide application frequencies, indicating the importance of limiting fungicide applications to preserve sensitivity. The genetic diversity of resistance mechanisms, reflected in the various mutations in the *β‐tubulin* gene, underscores the need for a deeper investigation into the fitness of the different genotypes to evaluate resistance spread in *P. viticola* populations. © 2025 The Author(s). *Pest Management Science* published by John Wiley & Sons Ltd on behalf of Society of Chemical Industry.

## Graphical abstract

Zoxamide resistance management in *Plasmopara viticola*: controlling selection pressure through optimal fungicide application frequency to reduce the spread of resistant strains possessing different point mutations associated with resistance.

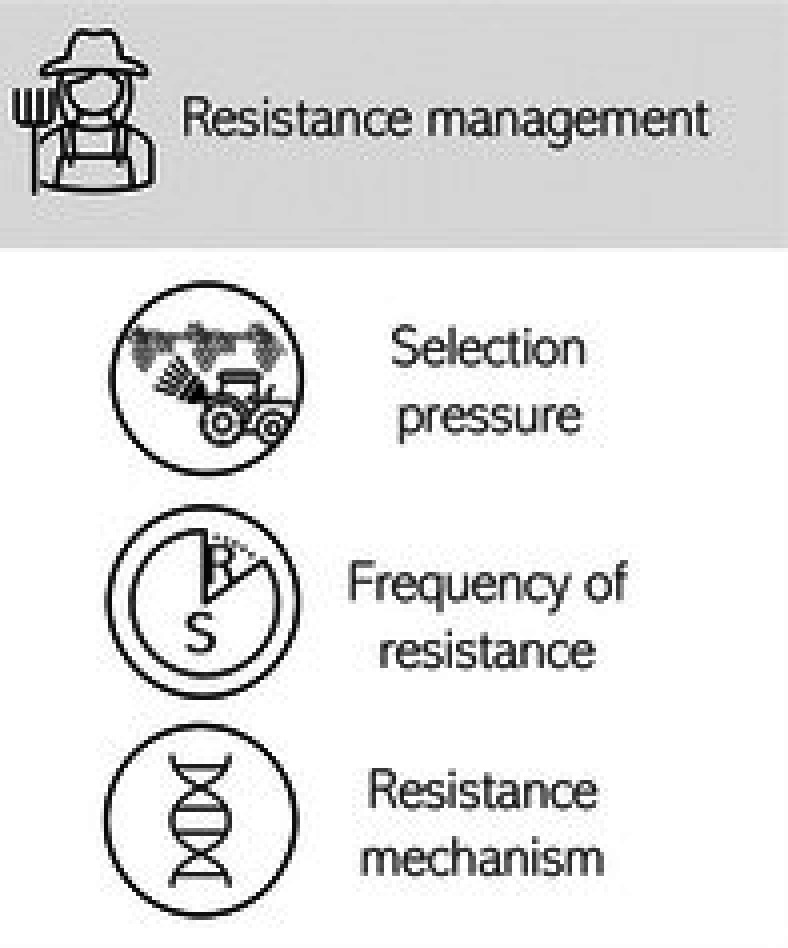

## 1 INTRODUCTION

Fungicides play a crucial role in managing grapevine downy mildew, and their effective utilization requires a comprehensive understanding of potential resistance outbreaks.

The causal agent of grapevine downy mildew, the oomycete *Plasmopara viticola* (Berk. *et* Curt.) Berlese & De Toni, is a biotrophic parasite and a polycyclic pathogen able to perform both sexual and asexual reproduction by forming oospores and zoospores in sporangia.^1^, ^2^ These characteristics contribute to its classification as a high‐risk pathogen for fungicide resistance.

The benzamide fungicide zoxamide is frequently employed in vineyards to manage downy mildew. It interferes with cytoskeleton and motor proteins of several species of oomycetes and ascomycetes (*e.g., Botrytis cinerea* Pers.), by inhibiting tubulin polymerization, causing mitotic arrest and disrupting cell division.^3^ It is classified by the Fungicide Resistance Action Committee (FRAC) as a low to medium risk for fungicide resistance. Indeed, resistance has been rarely found in field isolates.^4^, ^5^, ^6^ Most of the studies on the mechanism of resistance focus on laboratory mutants, and very little information is available in the literature concerning resistance to zoxamide in *P. viticola*. Only recently, the C239S mutation in *β‐tubulin* gene, previously identified in laboratory mutants of the oomycete *Phytophthora sojae* Kaufm. & Gerd,^7^ has been reported in *P. viticola* strains isolated from field populations.^8^, ^9^

The aim of this study was to gather information on the distribution and characterization of zoxamide resistance in *P. viticola* to support resistance management strategies. To achieve this goal, a twofold approach was adopted: (i) detecting and quantifying resistance across different selection pressure levels in Italian regions using a combined set of toxicological parameters (EC₅₀, EC₉₅, and MIC), and (ii) investigating the molecular basis of resistance through the characterization of mutations in the *β‐tubulin* gene.

A total of 126 samples, collected predominantly from the North‐Eastern Italian regions, were tested for zoxamide resistance over a 6‐year program spanning from 2017 to 2022. Sensitivity tests were primarily conducted on oospores to quantify resistant individuals within the population and gain insights into the effect of fungicide use, providing useful information for resistance management.^10^ Oospores are mainly formed at the end of the growing season by sexual reproduction, involving two mating types.^11^, ^12^ This characteristic makes them particularly suitable for assessing the effect of selection pressure exerted by fungicide applications. Additionally, since oospores represent the primary source of inoculum for the following season, their sensitivity profile provides valuable insights into the potential risk of resistant strain emergence in subsequent epidemics. After completing the oospore monitoring phase, 18 resistant strains were furthermore isolated from *P. viticola* sporangia and characterized for the mutations associated with resistance by sequencing a 720 bp portion of *β‐tubulin* gene to get insight on the resistance mechanism.

## 2 MATERIALS AND METHODS

### 2.1 Vineyards sampled

A total of 126 samples were collected at the end of the grapevine growing season (September–October) from 57 different vineyards over a 6‐year period (2017–2022). Most of the fields (118) are commercial vineyards, and only eight were used for experimental trials. The complete list of the samples, with information on the vineyard location, cultivar, number of zoxamide treatments applied within the season and type of vineyard (commercial or experimental), is available in Supporting Information, Table S1. Most of the samples were collected from the North‐Eastern Italian regions, namely Veneto (56 samples), Trentino‐Alto Adige (37 samples), Friuli‐Venezia Giulia (16 samples) and Emilia‐Romagna (three samples). Twelve samples were collected in the North‐Western Italy (11 samples in Lombardy, one sample in Piedmont) and only two samples were collected in the Southern Region Campania. Three‐quarters of the samples were collected from cv Glera (32.5%), used for producing Prosecco wine, and from international varieties such as Pinot gris (17.5%), Merlot (15.9%) and Chardonnay (12.7%). Local varieties covered the remaining samples.

The samples were collected from vineyards subjected to varying levels of selection pressure, determined by the frequency of zoxamide treatments performed during grapevine growing seasons. For 20 samples, no information could be obtained regarding the number of zoxamide applications performed by the farmers. Out of the remaining 106 samples, four were collected from vineyards untreated with zoxamide, three from vineyards treated with a single application of zoxamide, 82 from vineyards treated two to four times with zoxamide, 14 from vineyards treated five times with zoxamide, and three from vineyards treated 8–10 times (experimental vineyards). In commercial vineyards, the fungicide was always applied in combination with a partner possessing a different mode of action. Treatments were performed by farmers using their own equipment, following the recommended label doses.

### 2.2 Sample preparation

Leaves displaying necrotic lesions were randomly sampled from each vineyard and transported to the laboratory in plastic bags. The leaves were stored at 5 °C until further processing. Necrotic lesions were examined under an optical stereomicroscope (Zeiss, Göttingen, DE), and areas rich in oospores were carefully excised using a razor blade. For each location, three nylon bags were prepared, each containing 50 leaf fragments cut from different leaves. These bags were then overwintered in controlled conditions (5 °C, 30% relative humidity) until sensitivity tests were conducted.

### 2.3 Sensitivity assays

#### 2.3.1 Sensitivity assays on the oospores

Sensitivity to zoxamide was assessed in February–April 2018–2023 through oospore germination assays at increasing concentrations of zoxamide adapting a previous protocol.^10^ Briefly, the oospores were isolated from the leaf tissues by using a glass Potter and resuspended in water following a double filtration on 100 and 45 μm nylon filters.^10^ For each of the three nylon bags, a total of 1200 oospores divided into three technical replicates of 400 oospores each were plated on 1% water‐agar (Agar Noble DIFCO Difco, Thermo Fisher Scientific, Rodano, Milano, IT) amended with zoxamide active ingredient (a.i.) at the following concentrations: 0–0.01 – 0.1 – 1 – 10 – 100 mg/L active ingredient (2017–2018); 0–0.2 – 2 – 20 – 200 – 400 mg/L active ingredient (2019); 0–0.01 – 0.1 – 1 – 10 – 100 – 200 mg/L active ingredient (2020–2022). During the study, the range of fungicide concentrations was progressively adjusted based on preliminary results. According to sensitivity monitoring guidelines, dose rates should cover the sensitive range, include a fully inhibitory rate, and allow for accurate assessment of sensitivity shifts.^13^ Initially, the selected concentrations effectively assessed the sensitivity of *P. viticola* populations. However, as the study progressed, higher concentrations were needed to fully inhibit germination in some populations and accurately determine the MIC value. From the third year onward, a concentration of 200 mg/L was introduced to evaluate whether oospores could germinate at levels exceeding the field dose of zoxamide (180 mg/L).

The number of germinated oospores was checked at 7, 10 and 14 days after incubation at 20 °C in the dark to calculate the germination percentage (G). Germination Inhibition percentage (GI) was calculated at each fungicide concentration with the formula $\mathrm{GI}=100-\left(\frac{G_{x}}{G_{0}}\times 100\right)$ where G_x_ is the germination percentage at zoxamide concentration x and G_0_ is the germination percentage on the untreated control.

GI values were used to determine the toxicological parameters EC₅₀, EC₉₅, and MIC, which are the standard toxicological parameters used to assess fungicide sensitivity, as described in paragraph 2.5.

The percentage of resistant oospores (RO) within oospore population (sample) was calculated at 100 mg/L by subtracting GI_100_ (*i.e*., the germination inhibition percentages at 100 mg/L zoxamide) to 100.

#### 2.3.2 Strain isolation and sensitivity characterization

**Figure 1:**
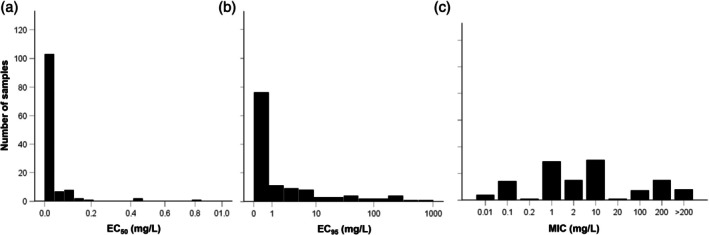
Frequency distribution of zoxamide EC_50_ (a), EC_95_ (b), and MIC (c) values (mg/L) for the 124 *P. viticola* samples.

Infected leaves, collected from the field (vineyards no. 27, 29, 30, and 57) in October 2023, were washed under running tap water to remove old sporulation and incubated overnight at 22 °C. Fresh sporulation was resuspended in sterile water the following day, and the resulting sporangial suspension was diluted to 5 × 10^4^ sporangia/mL before being sprayed with an airbrush onto leaf discs (13 mm diameter; *Vitis vinifera* L. cv Merlot) that had been pre‐treated with 100 mg/L of zoxamide and placed with abaxial face side up in Petri dishes containing moistened filter paper. After 1 week of incubation in a growth chamber at 22 °C under a 12:12 h light–dark photoperiod, strains were isolated from single sporangia following a serial dilution method.^14^ The strains were classified as resistant based on a discriminatory threshold of 100 mg/L zoxamide. Specifically, isolates capable of growing at this concentration were considered resistant. This threshold was selected based on the distribution of EC_95_ (Fig. 1(b)) and MIC values (Fig. 1(c)) obtained with oospore samples. The EC_95_ histogram shows that most samples have values well below this threshold, with only a small subset reaching or exceeding 100 mg/L. Similarly, the MIC distribution highlights a distinct group of samples with MIC values of 100 mg/L or higher, further supporting the choice of this cutoff to define resistance. Additionally, recent literature indicates that resistance in *P. viticola* isolates occurs at zoxamide concentrations exceeding 10 mg/L.^8^, ^9^ A total of 18 resistant strains were isolated: three from vineyard no. 27 (MarR1, MarR6, MarR11), one from vineyard no. 29 (CazR6), four from no. 30 (PasR1, PasR4, PasR9, PasR10), four from vineyard no. 47 (TesR2, TesR6, TesR9, TesR12), two from vineyard no. 48 (CasTNT11, CasTNT17), and four from vineyard no. 57 (GheR4, GheR5, GheR6, GheR9). The strains were weekly propagated on an increasing number of fresh leaves to collect sporangia for DNA extraction. Due to the low number of sporangia obtained during the initial propagation cycles, a total of 10 cycles were required to obtain sufficient biological material for DNA extraction. After 10 propagation cycles, a leaf disc assay with the discriminatory concentration of 100 mg/L of zoxamide was performed, confirming that the initial resistance phenotype remained unchanged. Two strains (GirI3 and GraI7) of the DiSAA culture collection showing EC_50_ < 0.01 mg/L and EC_95_ and MIC≤1 mg/L were used as sensitive reference.

### 2.4 *Β‐Tubulin* sequencing

Sequencing of a 720 bp portion of the *P. viticola β‐tubulin* gene was performed on all *P. viticola* isolates. The primers were designed with the NCBI Primer‐BLAST tool between nt 429 682–430 402 of the whole genome sequence of the pathogen MBPM02000031.1 retrieved from the NCBI database. The portion of *P. viticola* genome corresponding to the location of C239S mutation^7^ was determined through a BLASTn analysis by using the *β‐tubulin* gene of *Phytophthora sojae* accession n. XM_009531627.1 as reference. The selected primers were Pv_Btub_F2 (CACCTACTCTGTTTGCCCGT) and Pv_Btub_R2 (TCGTCCATACCCTCACCAGT). The primers were synthesized by Eurofins Genomics (Eurofins Genomics Europe Shared Services GmbH, Ebersberg, DE).

The DNA was extracted from sporangia following a previously described protocol,^15^ resuspended in 40 μL of TE buffer and spectrophotometrically checked for quantity and quality (Nanodrop ND1000; Thermo Fisher Scientific, Rodano, Milan, Italy). PCR amplification was performed in a 25 μL reaction mixtures containing 2 μL of DNA, 2.5 μL of Pv_Btub_F2 and Pv_Btub_R2 (5 μM), 5.5 μL of nuclease‐free water, and 12.5 μL of DreamTaq green PCR Master Mix (Thermo Fisher Scientific) using the Eppendorf Mastercycler Vapo.protect (Eppendorf, Milan, Italy) thermal cycler. The cycling conditions were: 10 min initial denaturation at 95 °C; 40 cycles of 15 s at 95 °C, 30 s at 59.1 °C, 30 s at 72 °C; and a final extension step at 72 °C for 5 min. Agarose gel electrophoresis was performed to verify the amplicons. Sanger sequencing was carried out by Eurofins Genomics. The sequence and electropherograms were analyzed using BioEdit Sequence Alignment Editor (Tom Hall, version 7.7.1) and FinchTV (Geospiza Inc., version 1.4.0), respectively. The genotypes of the strains were predicted based on the sequence at codon 239 as follows: CC, when the strain was homozygous for TGC, which encodes the amino acid cysteine (C); SS, when the strain was homozygous for either AGC or TCC, both of which encode the amino acid serine (S); CG, when the strain was heterozygous at nucleotide position 715, with TGC encoding cysteine (C) and GGC encoding glycine (G); SG, when the strain was heterozygous at nucleotide position 715, with AGC encoding serine (S) and GGC encoding glycine (G); CT, when the strain was heterozygous at nucleotide position 715, with TGC encoding cysteine (C) and ACC encoding threonine (T).

### 2.5 Data analysis

The EC_50_ and EC_95_ values, representing the fungicide concentrations inhibiting the oospore germination by 50 and 95% compared to the control, were estimated by using Probit Analysis (SPSS Statistics v. 29, IBM Milano, IT). MIC (Minimum Inhibitory Concentration) was defined as the lowest experimental concentration of zoxamide that completely prevented the oospore germination. The existence of correlation between toxicological parameters and zoxamide application in vineyard and between MIC and year of sampling was assessed through Kendall τ and Spearman ρ non‐parametric tests (SPSS v. 29).

A two‐sample t‐test was conducted to identify significant differences in EC_50_ and EC_95_ values between vineyards treated with zoxamide fewer than four times and those treated four or more times. Data were log_10_‐transformed to meet assumptions of normality and homogeneity of variances. Normality was assessed for both groups using the Shapiro–Wilk test, and homogeneity of variances was evaluated with Levene's test. Due to the lack of homogeneity of variances for EC_50_, a Welch's two‐sample t‐test was applied; for EC_95_, a standard two‐sample t‐test was used. Only vineyards with precisely estimated EC_50_ and EC_95_ values were included in the analysis, thus excluding values expressed as ranges (<0.01, <0.1, <0.2, 1–10, 10–100, 100–200, >200).

## 3 RESULTS

### 3.1 Sensitivity to zoxamide in oospore samples

The average values of G, GI, RO (%), EC_50_ and EC_95_, and MIC (mg/L) of all the samples are reported in Supporting Information, Table S2. The oospore germination (G) on the control medium ranged from 0.1 to 14.3% with an average value of 2.2% and a median of 1.3% (Supporting Information, Fig. S1). Only 10 samples (no. 8, 45, 46, 47, 48, 51, 52, 53, 54, 55) showed G values ranging from 6 to 14%.

The GI values showed a significant positive correlation (Tau Kendall = 0.538; Rho Spearman = 0,659; *N* = 693; *P* < 0.001) with increasing fungicide concentrations. The GI values of individual samples were suitable for calculating the toxicological parameters except for samples no. 110 and 113, where GI did not follow the typical dose–response curve.^16^ These samples were therefore excluded from the analyses.

The frequency distribution of EC₅₀ (Fig. 1(a)) and EC₉₅ (Fig. 1(b)) values for the analyzed *P. viticola* samples shows a strong skew toward lower concentrations, with only a few samples exhibiting higher values. Most samples had EC₅₀ values between 0.001 and 0.2 mg/L, while only three reached 0.4 and 0.8 mg/L. Similarly, the distribution of EC₉₅ values was highly skewed (Fig. 1(b)). The mean EC_95_ value was 11 mg/L, while the 95th percentile reached 26 mg/L, indicating that only 5% of the samples had values above this threshold. Only a small subset of eight isolates required concentrations above 100 mg/L for 95% germination inhibition. MIC values (Fig. 1(c)) showed a broader distribution, with most samples (74) falling in the 1–10 mg/L range and a small subgroup (23) requiring concentrations above 100 mg/L.

No significant correlation was found between EC_50_ and EC_95_/MIC (*P* > 0.85), whereas a significant correlation was observed between EC_95_ and MIC (*P* < 0.001).

### 3.2 Effect of zoxamide application *on P. viticola* populations

**Figure 2:**
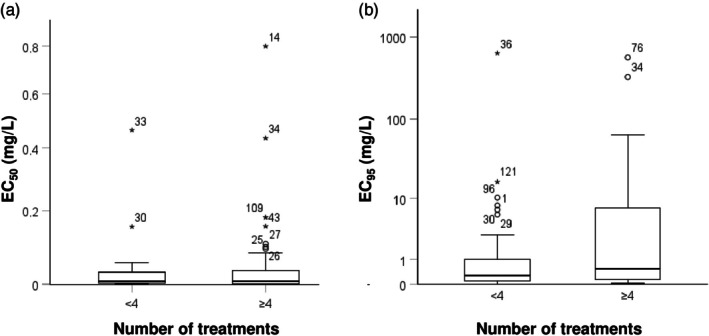
Box‐plot distribution of the average EC_50_ (a) and EC_95_ (b) values (mg/L) for samples grouped by the number of zoxamide applications in the vineyard (<4 or ≥4 applications).

EC_50_ values (Fig. 2(a)) did not differ based on the number of fungicide applications (*P* > 0.9). In contrast, the EC_95_ values showed a wider distribution in samples treated four or more times with zoxamide (Fig. 2(b)). EC_95_ values were significantly higher, by a factor of two, in these populations (*P* < 0.05).

RO values were generally below 12%, indicating that very few oospores were able to germinate at 100 mg/L zoxamide. Higher RO values (ranging between 18% and 33%) were observed only in samples no. 105, 108 and 109.

### 3.3 Molecular characterization of the strains

The sequences of all the strains are provided in Supporting Information, Table S3.

The electropherograms of the *β‐tubulin* region showed several synonymous mutations at codons 225, 256, 273, 284, 331 and 389. At codon 225, CTG was substituted by TTG in 16 strains (eight heterozygous and eight homozygous), both codons encoding leucine. At codon 256, AAT replaced AAC in 16 strains (eight heterozygous and eight homozygous), both encoding asparagine. Codon 273 exhibited TTG instead of CTG in 16 strains (eight heterozygous and eight homozygous), with both codons encoding leucine. Similarly, codon 284 showed TTG in place of TTA in 17 strains (nine heterozygous and eight homozygous), both encoding leucine. Codon 331 showed a CTT to CTA substitution, observed exclusively in the heterozygous GirI3 strain, with both codons encoding leucine. Lastly, at codon 389, TTT replaced TTC in 16 strains (eight heterozygous and eight homozygous), both codons encoding phenylalanine.

**Figure 3:**
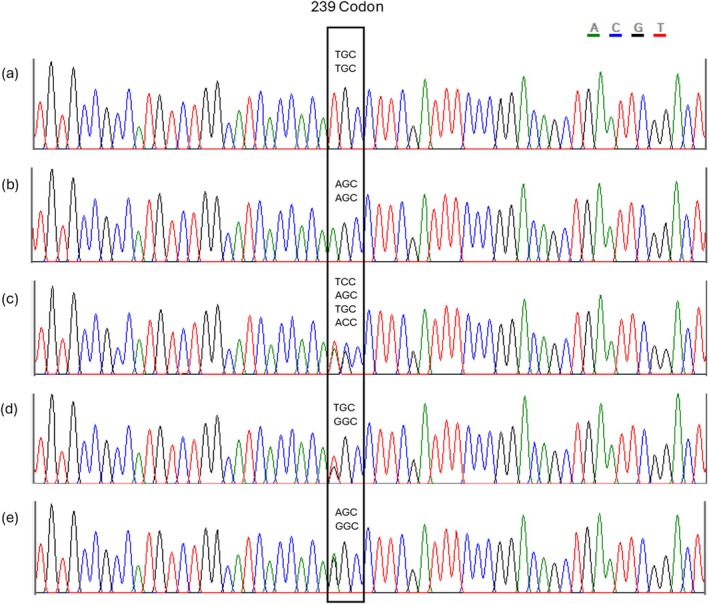
Electropherograms of the *β‐tubulin* region spanning codons 230–249 of the sensitive strain GirI3 (a), and the resistant strains GheR5 (b), MarR11 (c), CasTNT11 (d) and MarR1 (e). The black box highlights the possible variants at codon 239.

Non‐synonymous mutations were found only at codon 239, which is reported in the literature as the site of the mutation conferring resistance in *P. viticola* (Table 1; Fig. 3). The sensitive strains GirI3 and GraI7 were both homozygous for the codon TGC (Fig. 3(a)), which encodes cysteine, an amino acid associated with sensitivity, and exhibited a CC predicted genotype. The resistant strains GheR5, GheR6, and GheR9 were homozygous for AGC (Fig. 3(b)), encoding serine, an amino acid associated with resistance, and showed an SS predicted genotype. Resistant strain MarR11 displayed heterozygosity at two nucleotide positions (715 and 716), with two SNPs at codon 239: adenine and thymine at position 715, and guanine and cytosine at position 716 (Fig. 3(c)). This heterogeneity complicates genotype prediction, which could possibly be SS (TCC/AGC combination) or CT (TGC/ACC combination). The resistant strains CasTNT11 and CasTNT17 exhibited a CG genotype, being heterozygous for TGC (encoding cysteine) and GGC (encoding glycine) (Fig. 3(d)). Most of the resistant strains carried both AGC (encoding serine) and GGC (encoding glycine) codons (Fig. 3(e)), resulting in a serine/glycine (SG) genotype.

**Table 1:** Sensitivity phenotype (S=sensitive; R=resistant) and predicted genotypes based on codon 239 of the *β‐tubulin* gene in *P. viticola* strains

Isolate	Phenotype	Codon 239	Aminoacid	Genotype
GirI3, GraI7	S	TGC	C	CC
GheR5, GheR6, GheR9	R	AGC	S	SS
MarR11	R	TGC/ACC or TCC/AGC	C/T or S/S	CT or SS
CasTNT11, CasTNT17	R	TGC/GGC	C/G	CG
MarR1, MarR6, CazR6, PasR1, PasR4, PasR9, PasR10, TesR2, TesR6, TesR9, TesR12, GheR4	R	AGC/GGC	S/G	SG

## 4 DISCUSSION

### 4.1 Sensitivity determination in oospore samples

Toxicological parameters such as EC_50_, EC_90/95_, and MIC are widely used to assess fungicide resistance.^17^, ^18^ While EC_50_ alone is commonly employed for individual isolates, it may not be sufficient for population‐level monitoring, particularly for obligate parasites like *P. viticola*, where isolating strains for routine analysis is challenging.^14^, ^19^ In this study, EC_50_, EC_95_ and MIC were analyzed, alone or in combination, to determine the sensitivity profiles of oospore populations. The lack of correlation between EC_50_ and EC_95_/MIC values observed suggested that relying solely on EC_50_ may underestimate resistance levels in field populations.

Therefore, to achieve a more accurate characterization of resistance, EC_50_, EC_95_ and MIC were considered together, as each parameter provides complementary information. EC_50_, for instance, primarily reflects the median response of the population,^16^ potentially overlooking resistant subpopulations within a sample. This was evident in several oospore populations where EC_95_ and MIC exceeded 100 mg/L, indicative of resistance, while EC_50_ remained low. Conversely, among the three populations with EC_50_ values above 0.4 mg/L, only one sample had EC_95_ and MIC values >100 mg/L, while the others were fully inhibited at 10 mg/L. MIC alone may also be insufficient to determine sensitivity, since it depends on the range of concentrations tested. In this study, several samples had MIC values >100 mg/L, suggesting resistance,^9^, ^20^ while their EC_95_ values remained below 10 mg/L, suggesting a sensitivity profile. These findings confirmed that no single parameter fully described population sensitivity, and that combining EC₅₀, EC₉₅, and MIC could provide a more accurate characterization of resistance.

Given these observations, classification criteria were established. Samples were classified as sensitive to zoxamide when EC_50_ values were below 0.2 mg/L and both EC_95_ and MIC values below 26 mg/L. Resistant samples were defined as those with EC_95_ > 26 mg/L and MIC ≥100 mg/L. These thresholds were chosen based on the data achieved in this study and are consistent with recent studies conducted on *P. viticola* sporangia, where sensitive isolates displayed EC_50_ < 0.1 mg/L and resistant phenotypes showed EC_50_ values exceeding 25 mg/L and MIC values higher than 100 mg/L.^8^, ^9^, ^20^ Based on these criteria, we identified a total of 13 resistant samples, one of which also exhibiting EC_50_ > 0.4 mg/L. The remaining samples were sensitive to the fungicide, indicating that resistance to zoxamide was relatively infrequent but present in the tested populations.

### 4.2 Effect of fungicide use on *P. viticola* populations

The monitoring activity performed over 6 years on a large number of samples indicated that applying zoxamide four or more times per season in the field doubled the average EC_95_ values of oospore populations compared to fields where zoxamide was used no more than three times per season. A recent study in northeastern Italy similarly reported a progressive shift toward MIC values exceeding 100 mg/L over a 6‐year period,^20^ further confirming that repeated fungicide use contributes to changes in pathogen sensitivity. Indeed, the majority of the resistant samples (77%) identified in this study were collected from fields treated four to five times with zoxamide, suggesting that intense selection pressure in the field may have favored the selection of resistant strains, which then underwent mating, generating resistant oospores.

Quantifying resistant individuals provided valuable insights into the impact of the fungicide use on the pathogen populations. Practical resistance occurs when the resistant subpopulation becomes predominant over the sensitive one.^21^ Examining the percentage of oospores able to germinate at the discriminatory concentration of 100 mg/L (RO) within resistant populations, it is possible to notice that in most cases (62%) the resistant subpopulation accounted for less than 10% of the total. However, in two cases (samples 108 and 109) the resistant subpopulation was substantially higher (24–33%), approaching the proportion of sensitive individuals. Given the role of oospores in pathogen survival and primary infection establishment, higher RO values may indicate an increased risk of resistant strains spreading in subsequent seasons. In such cases, particular attention should be given to anti‐resistance strategies to prevent further selection of resistant strains, especially under conditions favoring multiple secondary cycles. It is well established that an increased number of fungicide applications per year accelerates the selection of resistant strains, both in cases of qualitative resistance (reference) and quantitative resistance.^22^ Reducing zoxamide applications could be an effective strategy for managing resistant strains. For other fungicide classes, such as QoIs, suspending treatments in the field has proven effective in restoring sensitivity within the oospore population.^23^ While this approach has not yet been evaluated for zoxamide, it could represent a viable strategy in fields where resistant oospores are present at high frequencies.

### 4.3 Resistance mechanism

Beside the number of fungicide applications performed in vineyard, the pathogen population sensitivity can be also influenced by other factors,^24^ among which is the resistance mechanism. In general, fungicide resistance is associated to different mechanisms (detoxification of the fungicide, overexpression of the target, exclusion or expulsion from the target site, alternative metabolic pathway and target site mutation) that can be found in fungal strains alone or in combination.^25^ For what concerns *P. viticola*, the known resistance mechanisms to members of QoIs, CAAs, QiI, QoSI and OSBPI classes are linked to point mutations in the genes encoding for the target proteins.^26^, ^27^, ^28^, ^29^, ^30^, ^31^ Recently, the mutation C239S/G in *β‐tubulin* gene was also associated with resistance to zoxamide in Italian isolates.^8^ In this study, the AGC codon encoding serine was found in homozygosis (SS genotype) in 11% of the resistant strains (SS genotype), but the predominant mutation (67% of the isolates) was C239S/G (SG genotype).

The resistant strain MArR11 showed a variable nucleotide composition at codon 239 of *β‐tubulin* gene. The possible genotypes could be SS or CT. Nevertheless, since the codon ACC (encoding threonine) has not been reported previously in the literature and has not been observed in any other strain, it is likely that the nucleotide combinations at codon 239 are TCC and AGC, both of which encode serine, and are associated with a SS genotype. However, this hypothesis cannot be verified within the context of this study and should be addressed in future studies, where homozygous strains with the different nucleotide compositions at codon 239 are obtained, tested for the sensitivity phenotype and then crossed to achieve different codon combinations and check the sensitivity profiles of the progeny.

The high variability at codon 239 of *β‐tubulin* genes, the only codon in the analyzed sequence showing non‐synonymous mutations, confirms the importance of the region in determining the sensitivity profile of *P. viticola* isolates. Future assessments of the fitness and spread of different genotypes would be valuable for determining the existence of fitness penalties and identifying point mutations more likely to pose challenges in the field. For instance, in the case of QoI resistance, the G143A mutation in the *cytb* gene is more commonly observed than the F129L mutation.^32^

## 5 CONCLUSION

This study provides new insights into zoxamide resistance in *P. viticola*, combining a quantitative evaluation of selection pressure on oospore populations with the molecular characterization of resistance‐associated mutations in sporangial isolates. The analysis of oospore populations revealed that resistance was generally rare and detected at low frequencies. A limited number of populations showed a substantial presence of resistant individuals. However, the increase in EC_95_ values in vineyards treated with zoxamide four or more times per season suggests that selection pressure can drive resistance development, emphasizing the need for careful fungicide management. These findings further confirm that repeated fungicide applications promote shifts in pathogen sensitivity, leading to a higher frequency of resistant strains in the sexual population. However, this effect was not observed in all vineyards, reinforcing the importance of site‐specific anti‐resistance strategies. Molecular analyses confirmed that resistance to zoxamide in *P. viticola* is associated with point mutations at codon 239 of the *β‐tubulin* gene, predominantly the C239S/G substitution, with the SG genotype being the most common. The high genetic variability observed at this codon underscores the need to further investigate its implications for resistance dynamics. Future studies should not only focus on fitness assessments to understand the persistence of resistant genotypes in field populations but also explore asexual structures of *P. viticola* to confirm these results and determine whether up to three zoxamide treatments per season have minimal impact on resistance spread.

## CONFLICT OF INTEREST

The authors declare that they have no known competing financial interests or personal relationships that could have appeared to influence the work reported in this article.

## Supporting information


**Data S1:** Supporting Information.

## Data Availability

The data that supports the findings of this study are available in the supplementary material of this article.
