# Supplementary material for: The role of selection pressure in shaping zoxamide resistance in Plasmopara viticola populations
Source: Pest Manag Sci. 2025 May 12;81(9):5360–7. doi: 10.1002/ps.8890 (PMC12332111; doi:10.1002/ps.8890)
Supplement: Supplementary file 1 — Data S1: Supporting Information. [file PS-81-5360-s001.docx]

**Supporting Information**

Table S1. List of the samples collected in 2017-2022 from the different vineyards with indication on the location (region, province and town), grapevine cultivar, number of zoxamide treatments performed during the growing season and type of vineyard (experimental or commercial).

| **Sample N** | **Vineyard N** | **Year of sampling** | **Region** | **Province** | **Town** | **Cultivar** | **Number of zoxamide treatments** | **Vineyard type** |
| --- | --- | --- | --- | --- | --- | --- | --- | --- |
| 1 | 31 | 2017 | Veneto | VR | Nogarole | Merlot | 3 | Commercial |
| 2 | 8 | 2017 | Trentino-Alto Adige | TN | Ravina, loc Masodema | Merlot | 2 | Commercial |
| 3 | 6 | 2017 | Veneto | TV | S. Vito di Valdobbiadene | Glera | 5 | Commercial |
| 4 | 49 | 2017 | Trentino-Alto Adige | TN | Volano | Marzemino | 3 | Commercial |
| 5 | 40 | 2017 | Trentino-Alto Adige | TN | Volano | Pinot grigio | 3 | Commercial |
| 6 | 16 | 2017 | Trentino-Alto Adige | TN | Nomi | Pinot grigio | 2 | Commercial |
| 7 | 18 | 2017 | Trentino-Alto Adige | TN | Nomi | Pinot grigio | 2 | Commercial |
| 8 | 21 | 2017 | Trentino-Alto Adige | TN | Roverè della luna | Schiava | 2 | Commercial |
| 9 | 2 | 2017 | Veneto | VR | Ronco all'Adige | Chardonnay | 0 | Experimental |
| 10 | 2 | 2017 | Veneto | VR | Ronco all'Adige | Chardonnay | 8 | Experimental |
| 11 | 2 | 2017 | Veneto | VR | Ronco all'Adige | Chardonnay | 5 | Experimental |
| 12 | 27 | 2017 | Veneto | TV | Valdobbiadene | Glera | 3 | Commercial |
| 13 | 25 | 2017 | Trentino-Alto Adige | TN | Pedersano | Traminer | 2 | Commercial |
| 14 | 34 | 2017 | Friuli-Venezia Giulia | PN | Sacile | Glera | 5 | Commercial |
| 15 | 19 | 2017 | Trentino-Alto Adige | TN | Roverè della luna | Chardonnay | 3 | Commercial |
| 16 | 14 | 2017 | Veneto | TV | Valdobbiadene | Glera | 4 | Commercial |
| 17 | 22 | 2017 | Friuli-Venezia Giulia | PN | Brugnera | Merlot | 5 | Commercial |
| 18 | 5 | 2017 | Friuli-Venezia Giulia | PN | Casarsa della Delizia | Pinot grigio | 2 | Commercial |
| 19 | 26 | 2017 | Friuli-Venezia Giulia | PN | Valvasone | Merlot | 4 | Commercial |
| 20 | 44 | 2017 | Veneto | TV | Valdobbiadene Vidor | Glera | 3 | Commercial |
| 21 | 1 | 2017 | Veneto | TV | Valdobbiadene | Glera | 3 | Commercial |
| 22 | 10 | 2018 | Veneto | VR | Salionze | Merlot | 4 | Commercial |
| 23 | 9 | 2018 | Lombardia | BS | Pozzolengo | Trebbiano di Lugana | 5 | Commercial |
| 24 | 7 | 2018 | Lombardia | MN | Castellaro Lagusello | Chardonnay | 4 | Commercial |
| 25 | 51 | 2018 | Veneto | VR | Valeggio Sul Mincio | Garganega | 5 | Commercial |
| 26 | 23 | 2018 | Veneto | VR | Valeggio Sul Mincio loc. Busetta | Pinot grigio | 4 | Commercial |
| 27 | 15 | 2018 | Veneto | VR | Valeggio Sul Mincio | Corvinone | 4 | Commercial |
| 28 | 8 | 2018 | Trentino-Alto Adige | TN | Ravina, loc. Masodema | Merlot | 4 | Commercial |
| 29 | 20 | 2018 | Trentino-Alto Adige | TN | Roverè della Luna | Pinot Grigio | 3 | Commercial |
| 30 | 32 | 2018 | Trentino-Alto Adige | TN | Laghetto Daone | Pinot Grigio | 3 | Commercial |
| 31 | 38 | 2018 | Trentino-Alto Adige | TN | Laghetto | Pinot Grigio |  | Commercial |
| 32 | 33 | 2018 | Trentino-Alto Adige | BZ | Winkel | Schiava | 3 | Commercial |
| 33 | 28 | 2018 | Trentino-Alto Adige | TN | Volano | Pinot Grigio | 2 | Commercial |
| 34 | 43 | 2018 | Trentino-Alto Adige | TN | Isera | Marzemino | 5 | Commercial |
| 35 | 17 | 2018 | Veneto | TV | Valdobbiadene | Glera | 4 | Commercial |
| 36 | 1 | 2018 | Veneto | TV | Valdobbiadene | Glera | 3 | Commercial |
| 37 | 47 | 2018 | Veneto | TV | Vidor | Glera | 4 | Commercial |
| 38 | 27 | 2018 | Veneto | TV | Valdobbiadene | Glera | 3 | Commercial |
| 39 | 48 | 2018 | Friuli-Venezia Giulia | PN | Casarsa della Delizia | Glera | 4 | Commercial |
| 40 | 6 | 2018 | Veneto | TV | S. Vito di Valdobbiadene | Glera | 5 | Commercial |
| 41 | 14 | 2018 | Veneto | TV | Valdobbiadene | Glera | 4 | Commercial |
| 42 | 52 | 2018 | Friuli-Venezia Giulia | UD | Ca' Bolani | Chardonnay | 4 | Commercial |
| 43 | 35 | 2018 | Friuli-Venezia Giulia | PN | Sacile | Merlot | 5 | Commercial |
| 44 | 10 | 2019 | Veneto | VR | Salionze | Merlot |  | Commercial |
| 45 | 9 | 2019 | Lombardia | BS | Pozzolengo | Trebbiano di Lugana |  | Commercial |
| 46 | 7 | 2019 | Lombardia | MN | Castellaro Lagusello | Chardonnay |  | Commercial |
| 47 | 54 | 2019 | Veneto | VR | Valeggio Sul Mincio | Garganega |  | Commercial |
| 48 | 23 | 2019 | Veneto | VR | Valeggio Sul Mincio loc. Busetta | Pinot grigio |  | Commercial |
| 49 | 15 | 2019 | Veneto | VR | Valeggio Sul Mincio | Corvinone | 3 | Commercial |
| 50 | 8 | 2019 | Trentino-Alto Adige | TN | Ravina, loc. Masodema | Merlot |  | Commercial |
| 51 | 20 | 2019 | Trentino-Alto Adige | TN | Roverè della Luna | Pinot Grigio | 2 | Commercial |
| 52 | 32 | 2019 | Trentino-Alto Adige | TN | Laghetto Daone | Pinot Grigio |  | Commercial |
| 53 | 38 | 2019 | Trentino-Alto Adige | TN | Laghetto | Pinot Grigio |  | Commercial |
| 54 | 33 | 2019 | Trentino-Alto Adige | BZ | Winkel | Schiava |  | Commercial |
| 55 | 28 | 2019 | Trentino-Alto Adige | TN | Volano | Pinot Grigio |  | Commercial |
| 56 | 43 | 2019 | Trentino-Alto Adige | TN | Isera | Marzemino | 5 | Commercial |
| 57 | 17 | 2019 | Veneto | TV | Valdobbiadene | Glera |  | Commercial |
| 58 | 1 | 2019 | Veneto | TV | Valdobbiadene | Glera | 3 | Commercial |
| 59 | 47 | 2019 | Veneto | TV | Vidor | Glera | 4 | Commercial |
| 60 | 27 | 2019 | Veneto | TV | Valdobbiadene | Glera |  | Commercial |
| 61 | 6 | 2019 | Veneto | TV | S. Vito di Valdobbiadene | Glera |  | Commercial |
| 62 | 14 | 2019 | Veneto | TV | Valdobbiadene | Glera | 4 | Commercial |
| 63 | 35 | 2019 | Friuli-Venezia Giulia | PN | Sacile | Merlot |  | Commercial |
| 64 | 7 | 2020 | Lombardia | MN | Castellaro Lagusello | Chardonnay | 3 | Commercial |
| 65 | 13 | 2020 | Veneto | VR | Valeggio Sul Mincio | Merlot | 4 | Commercial |
| 66 | 23 | 2020 | Veneto | VR | Valeggio Sul Mincio loc Busetta | Pinot grigio | 3 | Commercial |
| 67 | 15 | 2020 | Veneto | VR | Valeggio Sul Mincio | Corvinone | 4 | Commercial |
| 68 | 8 | 2020 | Trentino-Alto Adige | TN | Ravina, loc. Masodema | Merlot | 5 | Commercial |
| 69 | 20 | 2020 | Trentino-Alto Adige | TN | Roverè della Luna | Pinot Grigio | 3 | Commercial |
| 70 | 32 | 2020 | Trentino-Alto Adige | TN | Laghetto Daone | Pinot Grigio | 3 | Commercial |
| 71 | 28 | 2020 | Trentino-Alto Adige | TN | Volano | Pinot Grigio | 2 | Commercial |
| 72 | 43 | 2020 | Trentino-Alto Adige | TN | Isera | Marzemino | 5 | Commercial |
| 73 | 1 | 2020 | Veneto | TV | Valdobbiadene | Glera | 4 | Commercial |
| 74 | 47 | 2020 | Veneto | TV | Vidor | Glera | 4 | Commercial |
| 75 | 27 | 2020 | Veneto | TV | Valdobbiadene | Glera | 4 | Commercial |
| 76 | 6 | 2020 | Veneto | TV | S. Vito di Valdobbiadene | Glera | 4 | Commercial |
| 77 | 14 | 2020 | Veneto | TV | Valdobbiadene | Glera | 4 | Commercial |
| 78 | 12 | 2020 | Veneto | TV | Motta di Livenza | Glera | 4 | Commercial |
| 79 | 24 | 2020 | Friuli-Venezia Giulia | PN | Fontanafredda Frazione Nave | Glera | 5 | Commercial |
| 80 | 35 | 2020 | Friuli-Venezia Giulia | PN | Sacile | Merlot | 5 | Commercial |
| 81 | 4 | 2020 | Emilia-Romagna | RA | Ravenna | Trebbiano | 0 | Commercial |
| 82 | 41 | 2020 | Emilia-Romagna | RA | Ravenna | Trebbiano | 1 | Commercial |
| 83 | 11 | 2020 | Emilia-Romagna | RA | Bagnacavallo | Trebbiano | 2 | Commercial |
| 84 | 38 | 2020 | Trentino-Alto Adige | TN | Laghetto | Pinot grigio | 3 | Commercial |
| 85 | 7 | 2021 | Lombardia | MN | Castellaro Lagusello | Chardonnay | 3 | Commercial |
| 86 | 13 | 2021 | Veneto | VR | Valeggio sul Mincio | Merlot | 4 | Commercial |
| 87 | 23 | 2021 | Veneto | VR | Valeggio sul Mincio loc Busetta | Pinot grigio | 4 | Commercial |
| 88 | 15 | 2021 | Veneto | VR | Valeggio sul Mincio | Corvinone | 4 | Commercial |
| 89 | 8 | 2021 | Trentino-Alto Adige | TN | Ravina, loc Masodema | Merlot | 2 | Commercial |
| 90 | 43 | 2021 | Trentino-Alto Adige | TN | Isera | Marzemino | 3 | Commercial |
| 91 | 1 | 2021 | Veneto | TV | Valdobbiadene | Glera | 3 | Commercial |
| 92 | 47 | 2021 | Veneto | TV | Vidor | Glera | 4 | Commercial |
| 93 | 27 | 2021 | Veneto | TV | Valdobbiadene | Glera | 4 | Commercial |
| 94 | 6 | 2021 | Veneto | TV | S. Vito di Valdobbiadene | Glera | 4 | Commercial |
| 95 | 14 | 2021 | Veneto | TV | Valdobbiadene | Glera | 4 | Commercial |
| 96 | 24 | 2021 | Friuli-Venezia Giulia | PN | Fontanafredda Frazione Nave | Glera | 3 | Commercial |
| 97 | 35 | 2021 | Friuli-Venezia Giulia | PN | Sacile | Merlot | 5 | Commercial |
| 98 | 3 | 2021 | Veneto | VR | Castelnuovo del Garda | Merlot | 0 | Experimental |
| 99 | 3 | 2021 | Veneto | VR | Castelnuovo del Garda | Merlot | 10 | Experimental |
| 100 | 3 | 2021 | Veneto | VR | Castelnuovo del Garda | Merlot | 10 | Experimental |
| 101 | 3 | 2021 | Veneto | VR | Castelnuovo del Garda | Merlot | 2 | Experimental |
| 102 | 53 | 2021 | Trentino-Alto Adige | TN | Rovereto loc Navesel | Chardonnay | 2 | Commercial |
| 103 | 37 | 2021 | Trentino-Alto Adige | TN | Romagnano Alveo | Pinot grigio | 3 | Commercial |
| 104 | 46 | 2021 | Trentino-Alto Adige | TN | Mattarello loc Acquaviva | Cabernet | 3 | Commercial |
| 105 | 36 | 2021 | Friuli-Venezia Giulia | PN | Roveredo in Piano | Glera | 4 | Commercial |
| 106 | 15 | 2021 | Veneto | VR | Valeggio sul Mincio | Corvinone | 2 | Commercial |
| 107 | 42 | 2022 | Trentino-Alto Adige | TN | Rovereto | Marzemino | 2 | Commercial |
| 108 | 1 | 2022 | Veneto | TV | Valdobbiadene | Glera | 4 | Commercial |
| 109 | 47 | 2022 | Veneto | TV | Vidor | Glera | 4 | Commercial |
| 110 | 27 | 2022 | Veneto | TV | Valdobbiadene | Glera | 4 | Commercial |
| 111 | 6 | 2022 | Veneto | TV | S. Vito di Valdobbiadene | Glera | 4 | Commercial |
| 112 | 14 | 2022 | Veneto | TV | Valdobbiadene | Glera | 4 | Commercial |
| 113 | 24 | 2022 | Friuli-Venezia Giulia | PN | Fontanafredda Frazione Nave | Glera | 4 | Commercial |
| 114 | 34 | 2022 | Friuli-Venezia Giulia | PN | Sacile | Glera | 4 | Commercial |
| 115 | 53 | 2022 | Trentino-Alto Adige | TN | Rovereto loc Navesel | Chardonnay | 2 | Commercial |
| 116 | 37 | 2022 | Trentino-Alto Adige | TN | Romagnano Alveo | Pinot grigio | 3 | Commercial |
| 117 | 46 | 2022 | Trentino-Alto Adige | TN | Mattarello loc Acquaviva | Cabernet | 1 | Commercial |
| 118 | 36 | 2022 | Friuli-Venezia Giulia | PN | Roveredo in Piano | Glera |  | Commercial |
| 119 | 39 | 2022 | Lombardia | BS | Adro 1 | Chardonnay | 2 | Commercial |
| 120 | 50 | 2022 | Lombardia | BS | Erbusco 3 | Chardonnay | 4 | Commercial |
| 121 | 45 | 2022 | Lombardia | BS | Iseo | Chardonnay | 2 | Commercial |
| 122 | 57 | 2022 | Piemonte | NO | Ghemme | Vespolina | 0 | Experimental |
| 123 | 55 | 2022 | Campania | AV | Avellino | Greco | 1 | Commercial |
| 124 | 30 | 2022 | Lombardia | BS | Passirano | Chardonnay | 3 | Commercial |
| 125 | 29 | 2022 | Lombardia | BS | Cazzago | Chardonnay | 3 | Commercial |
| 126 | 56 | 2022 | Campania | SA | Battipaglia | Greco |  | Commercial |

Table S2. Germination percentages in absence of fungicide (G), germination inhibition percentages (GI) at each fungicide concentrations (0.01-200 mg/L), percentages of resistant oospores (RO) at 100 mg/L zoxamide, average EC_50_ and EC_95_ (mg/L) values, and MIC values (mg/L) of the analyzed samples.

| **Sample N** | **G (%)** | **GI (%)** | | | | | | | | | | **RO (at 100 mg/L)** | **EC_50_ (mg/L)** | **EC_95_ (mg/L)** | **MIC (mg/L)** |
| --- | --- | --- | --- | --- | --- | --- | --- | --- | --- | --- | --- | --- | --- | --- | --- |
|  |  | **0.01 mg/L** | **0.1 mg/L** | **0.2 mg/L** | **1 mg/L** | **2 mg/L** | **10 mg/L** | **20 mg/L** | **100 mg/L** | **200 mg/L** | **400 mg/L** |  |  |  |  |
| 1 | 2.1 | 11.8 | 73.5 | - | 95.6 |  | 91.2 | - | 95.6 | - | - | 4.4 | 0.056 | 8 | >100 |
| 2 | 0.9 | 81.8 | 90.9 | - | 100 |  | 100 | - | 100 | - | - | 0 | 0.001 | 0.127 | 1 |
| 3 | 0.9 | 100 | 85.8 | - | 100 |  | 100 | - | 100 | - | - | 0 | <0.01 | 0.037 | 1 |
| 4 | 0.5 | 71.5 | 85.8 | - | 100 |  | 100 | - | 100 | - | - | 0 | 0.003 | 0.248 | 1 |
| 5 | 0.1 | 0 | 100 | - | 100 |  | 100 | - | 100 | - | - | 0 | <0.1 | <0.1 | 0.1 |
| 6 | 0.1 | 100 | 100 | - | 100 |  | 100 | - | 100 | - | - | 0 | <0.01 | <0.01 | 0.01 |
| 7 | 0.4 | 74 | 74 | - | 100 |  | 100 | - | 100 | - | - | 0 | 0.002 | 0.612 | 1 |
| 8 | 7.2 | 50.7 | 88.6 | - | 93.7 |  | 97.5 | - | 97.5 | - | - | 2.5 | 0.004 | 3 | >100 |
| 9 | 1.7 | 61.4 | 72.4 | - | 94.5 |  | 100 | - | 100 | - | - | 0 | 0.006 | 1 | 10 |
| 10 | 0.5 | 100 | 100 | - | 100 |  | 100 | - | 100 | - | - | 0 | <0.01 | <0.01 | 0.01 |
| 11 | 0.5 | 41.2 | 41.2 | - | 60.8 |  | 100 | - | 100 | - | - | 0 | 0.08 | 26 | 10 |
| 12 | 1 | 100 | 91.5 | - | 100 |  | 100 | - | 100 | - | - | 0 | <0.01 | 0.005 | 1 |
| 13 | 0.2 | 66.3 | 100 | - | 100 |  | 100 | - | 100 | - | - | 0 | 0.007 | 0.025 | 0.1 |
| 14 | 0.1 | 17.2 | 15.7 | - | 18.2 |  | 100 | - | 100 | - | - | 0 | 0.8 | 7.4 | 10 |
| 15 | 0.9 | 28.3 | 73.1 | - | 82.1 |  | 100 | - | 100 | - | - | 0 | 0.04 | 2.8 | 10 |
| 16 | 0.7 | 100 | 86.6 | - | 100 |  | 100 | - | 100 | - | - | 0 | <0.01 | 0.03 | 1 |
| 17 | 0.8 | 77.5 | 78.5 | - | 88.8 |  | 100 | - | 100 | - | - | 0 | 0.001 | 2 | 10 |
| 18 | 0.5 | 65.8 | 82.9 | - | 100 |  | 100 | - | 100 | - | - | 0 | 0.004 | 0.317 | 1 |
| 19 | 1.5 | 87.2 | 100 | - | 100 |  | 100 | - | 100 | - | - | 0 | 0.004 | 0.016 | 0.1 |
| 20 | 0.8 | 87.7 | 100 | - | 100 |  | 100 | - | 100 | - | - | 0 | 0.003 | 0.015 | 0.1 |
| 21 | 4.4 | 98.1 | 98.1 | - | 100 |  | 100 | - | 100 | - | - | 0 | <0.01 | 0.002 | 1 |
| 22 | 1.42 | 29.6 | 71.8 | - | 100 |  | 100 | - | 100 | - | - | 0 | 0.028 | 0.459 | 1 |
| 23 | 1.24 | 54 | 87.1 | - | 100 |  | 100 | - | 100 | - | - | 0 | 0.009 | 0.217 | 1 |
| 24 | 1.3 | 60.8 | 66.2 | - | 94.6 |  | 100 | - | 100 | - | - | 0 | 0.008 | 2 | 10 |
| 25 | 1.32 | 0 | 62.9 | - | 93.9 |  | 100 | - | 100 | - | - | 0 | 0.092 | 0.696 | 10 |
| 26 | 0.47 | 14.9 | 31.9 | - | 100 |  | 100 | - | 100 | - | - | 0 | 0.097 | 1 | 1 |
| 27 | 3.24 | 3.1 | 63.3 | - | 89.5 |  | 94.8 | - | 100 | - | - | 0 | 0.105 | 3 | 100 |
| 28 | 1.87 | 28.9 | 86.6 | - | 95.7 |  | 100 | - | 100 | - | - | 0 | 0.023 | 0.436 | 10 |
| 29 | 1.31 | 69.5 | 56.5 | - | 87.8 |  | 100 | - | 100 | - | - | 0 | 0.006 | 6 | 10 |
| 30 | 1.3 | 16.9 | 36.2 | - | 74.6 |  | 100 | - | 100 | - | - | 0 | 0.153 | 7 | 10 |
| 31 | 1.36 | 30.9 | 100 | - | 100 |  | 100 | - | 100 | - | - | 0 | 0.015 | 0.065 | 0.1 |
| 32 | 2.95 | 57.6 | 69.2 | - | 100 |  | 100 | - | 100 | - | - | 0 | 0.01 | 0.728 | 1 |
| 33 | 0.7 | 28.6 | 0 | - | 12.9 |  | 100 | - | 100 | - | - | 0 | 0.463 | 1-10 | 10 |
| 34 | 1.55 | 5.8 | 52.9 | - | 58.7 |  | 76.8 | - | 88.4 | - | - | 11.6 | 0.435 | 325 | >100 |
| 35 | 1.58 | 60.1 | 50 | - | 85.4 |  | 100 | - | 100 | - | - | 0 | 0.015 | 7 | 1 |
| 36 | 2.4 | 62.9 | 81.7 | - | 89.2 |  | 89.2 | - | 89.2 | - | - | 10.8 | 0.001 | 631 | >100 |
| 37 | 1.89 | 46.6 | 90.5 | - | 51.3 |  | 90.5 | - | 100 | - | - | 0 | 0.009 | 1 | 100 |
| 38 | 0.76 | 11.8 | 88.2 | - | 100 |  | 100 | - | 100 | - | - | 0 | 0.032 | 0.156 | 1 |
| 39 | 2.47 | 74.5 | 100 | - | 100 |  | 100 | - | 100 | - | - | 0 | 0.006 | 0.022 | 0.1 |
| 40 | 2.71 | 73.8 | 77.1 | - | 100 |  | 100 | - | 100 | - | - | 0 | 0.002 | 0.493 | 1 |
| 41 | 2.47 | 14.2 | 72.5 | - | 79.4 |  | 89.9 | - | 96.8 | - | - | 3.2 | 0.074 | 19 | >100 |
| 42 | 0.69 | 50.7 | 37.7 | - | 50.7 |  | 100 | - | 100 | - | - | 0 | 0.074 | 63 | 10 |
| 43 | 0.36 | 0 | 25 | - | 100 |  | 100 | - | 100 | - | - | 0 | 0.154 | 0.422 | 1 |
| 44 | 4.3 | - | - | 100 | - | 100 | - | 100 | - | 100 | 100 | - | <0.2 | <0.2 | 0.2 |
| 45 | 14.3 | - | - | 98.3 | - | 100 | - | 100 | - | 100 | 100 | - | 0.007 | 0.097 | 2 |
| 46 | 7.1 | - | - | 96.5 | - | 100 | - | 100 | - | 100 | 100 | - | 0.006 | 0.154 | 2 |
| 47 | 6.3 | - | - | 88 | - | 97.4 | - | 97.44 | - | 100 | 100 | - | 0.001 | 1 | 200 |
| 48 | 6.6 | - | - | 97.6 | - | 100 | - | 100 | - | 100 | 100 | - | 0.003 | 0.101 | 2 |
| 49 | 3.4 | - | - | 92.7 | - | 100 | - | 100 | - | 100 | 100 | - | 0.03 | 0.266 | 2 |
| 50 | 4.3 | - | - | 98.1 | - | 100 | - | 100 | - | 100 | 100 | - | 0.011 | 0.109 | 2 |
| 51 | 14.1 | - | - | 96.4 | - | 100 | - | 100 | - | 100 | 100 | - | 0.006 | 0.159 | 2 |
| 52 | 6.7 | - | - | 100 | - | 100 | - | 100 | - | 100 | 100 | - | <0.2 | <0.2 | 2 |
| 53 | 12.9 | - | - | 98.1 | - | 100 | - | 100 | - | 100 | 100 | - | 0.011 | 0.109 | 2 |
| 54 | 7 | - | - | 97.7 | - | 100 | - | 100 | - | 100 | 100 | - | 0.002 | 0.096 | 2 |
| 55 | 7.3 | - | - | 97.8 | - | 100 | - | 100 | - | 100 | 100 | - | 0.002 | 0.092 | 2 |
| 56 | 4.8 | - | - | 100 | - | 100 | - | 100 | - | 100 | 100 | - | <0.2 | <0.2 | 2 |
| 57 | 4.3 | - | - | 92.2 | - | 100 | - | 100 | - | 100 | 100 | - | 0.033 | 0.274 | 2 |
| 58 | 2.1 | - | - | 96.2 | - | 96.2 | - | 100 | - | 100 | 100 | - | <0.2 | 0.213 | 20 |
| 59 | 2.2 | - | - | 88.4 | - | 92.6 | - | 96.16 | - | 100 | 100 | - | 0.001 | 3 | 200 |
| 60 | 0.9 | - | - | 91.2 | - | 100 | - | 100 | - | 100 | 100 | - | 0.039 | 0.288 | 2 |
| 61 | 2.8 | - | - | 91.2 | - | 85.5 | - | 97.07 | - | 100 | 100 | - | 0.001 | 5 | 200 |
| 62 | 1.3 | - | - | 93.8 | - | 100 | - | 100 | - | 100 | 100 | - | 0.023 | 0.247 | 2 |
| 63 | 1.2 | - | - | 100 | - | 100 | - | 100 | - | 100 | 100 | - | <0.2 | <0.2 | 2 |
| 64 | 3.7 | 47.7 | 88.6 | - | 90.9 | - | 97.7 | - | 100 | 100 | - | 0 | 0.008 | 1 | 100 |
| 65 | 2.9 | 77.1 | 91.4 | - | 94.3 | - | 100 | - | 100 | 100 | - | 0 | 0.001 | 0.42 | 10 |
| 66 | 1.9 | 47.8 | 82.6 | - | 91.3 | - | 100 | - | 100 | 100 | - | 0 | 0.011 | 1 | 10 |
| 67 | 0.9 | 54.5 | 72.7 | - | 90.9 | - | 90.9 | - | 90.9 | 100 | - | 9.1 | 0.003 | 44 | 200 |
| 68 | 0.3 | 0 | 100 | - | 100 | - | 100 | - | 100 | 100 | - | 0 | 0.035 | 0.12 | 0.01 |
| 69 | 2.4 | 65.5 | 93.1 | - | 96.6 | - | 100 | - | 100 | 100 | - | 0 | 0.003 | 0.281 | 10 |
| 70 | 3.7 | 61.4 | 90.9 | - | 93.2 | - | 100 | - | 100 | 100 | - | 0 | 0.003 | 0.594 | 10 |
| 71 | 1.6 | 78.9 | 78.9 | - | 94.7 | - | 100 | - | 100 | 100 | - | 0 | 0.001 | 0.927 | 10 |
| 72 | 2.3 | 66.7 | 66.7 | - | 77.8 | - | 96.3 | - | 96.3 | 100 | - | 3.7 | 0.003 | 27 | 200 |
| 73 | 0.9 | 63.6 | 100 | - | 100 | - | 100 | - | 100 | 100 | - | 0 | 0.007 | 0.037 | 0.1 |
| 74 | 2.3 | 81.5 | 96.3 | - | 100 | - | 100 | - | 100 | 100 | - | 0 | 0.001 | 0.059 | 1 |
| 75 | 2 | 62.5 | 95.8 | - | 100 | - | 100 | - | 100 | 100 | - | 0 | 0.006 | 0.084 | 1 |
| 76 | 3.1 | 51.4 | 59.5 | - | 62.2 | - | 78.4 | - | 94.6 | 97.3 | - | 5.4 | 0.024 | 562 | >200 |
| 77 | 1.2 | 57.1 | 92.9 | - | 92.9 | - | 100 | - | 100 | 100 | - | 0 | 0.004 | 0.552 | 10 |
| 78 | 1.1 | 38.5 | 61.5 | - | 100 | - | 100 | - | 100 | 100 | - | 0 | 0.027 | 0.824 | 1 |
| 79 | 1.5 | 55.6 | 100 | - | 100 | - | 100 | - | 100 | 100 | - | 0 | 0.009 | 0.043 | 0.1 |
| 80 | 0.8 | 77.8 | 88.9 | - | 100 | - | 100 | - | 100 | 100 | - | 0 | 0.001 | 0.172 | 1 |
| 81 | 0.7 | 25 | 62.5 | - | 87.5 | - | 100 | - | 100 | 100 | - | 0 | 0.05 | 2 | 10 |
| 82 | 0.6 | 42.9 | 100 | - | 100 | - | 100 | - | 100 | 100 | - | 0 | 0.012 | 0.053 | 0.1 |
| 83 | 0.5 | 66.7 | 83.3 | - | 100 | - | 100 | - | 100 | 100 | - | 0 | 0.004 | 0.306 | 1 |
| 84 | 0.5 | 33.3 | 100 | - | 100 | - | 100 | - | 100 | 100 | - | 0 | 0.015 | 0.062 | 0.1 |
| 85 | 0.3 | 75 | 100 | - | 75 | - | 100 | - | 100 | 100 | - | 0 | <0.01 | 3 | 10 |
| 86 | 0.7 | 88 | 88 | - | 88 | - | 100 | - | 100 | 100 | - | 0 | <0.01 | 2.5 | 100 |
| 87 | 0.4 | 47 | 80 | - | 100 | - | 100 | - | 100 | 100 | - | 0 | 0.013 | 0.4 | 1 |
| 88 | 1.3 | 87 | 100 | - | 93 | - | 100 | - | 100 | 100 | - | 0 | <0.01 | 0.1 | 10 |
| 89 | 0.7 | 88 | 88 | - | 100 | - | 100 | - | 100 | 100 | - | 0 | <0.01 | 0.2 | 1 |
| 90 | 2.3 | 70 | 96 | - | 96 | - | 100 | - | 100 | 100 | - | 0 | 0.002 | 0.2 | 10 |
| 91 | 0.9 | 91 | 100 | - | 91 | - | 82 | - | 91 | 100 | - | 9 | <0.01 | 100-200 | 200 |
| 92 | 1.1 | 54 | 85 | - | 92 | - | 100 | - | 92 | 100 | - | 8 | 0.002 | 7.3 | 200 |
| 93 | 0.5 | 83 | 100 | - | 100 | - | 100 | - | 100 | 100 | - | 0 | 0.003 | 0.02 | 0.1 |
| 94 | 1.6 | 95 | 95 | - | 84 | - | 100 | - | 95 | 100 | - | 5 | <0.01 | 2.3 | 200 |
| 95 | 1.9 | 91 | 87 | - | 87 | - | 96 | - | 100 | 100 | - | 0 | <0.01 | 2.8 | 100 |
| 96 | 0.3 | 50 | 50 | - | 75 | - | 100 | - | 100 | 100 | - | 0 | 0.032 | 10.2 | 10 |
| 97 | 0.4 | 80 | 60 | - | 100 | - | 80 | - | 100 | 100 | - | 0 | <0.01 | 36.4 | 100 |
| 98 | 0.3 | 50 | 75 | - | 100 | - | 100 | - | 100 | 100 | - | 0 | 0.013 | 0.5 | 1 |
| 99 | 1.5 | 94 | 94 | - | 94 | - | 94 | - | 100 | 100 | - | 0 | <0.01 | 0.2 | 100 |
| 100 | 0.7 | 100 | 100 | - | 88 | - | 100 | - | 100 | 100 | - | 0 | <0.01 | 0.014 | 10 |
| 101 | 0.4 | 100 | 80 | - | 80 | - | 100 | - | 100 | 100 | - | 0 | <0.01 | 4.2 | 10 |
| 102 | 1.1 | 77 | 100 | - | 100 | - | 100 | - | 100 | 100 | - | 0 | 0.005 | 0.03 | 0.1 |
| 103 | 1.8 | 95 | 90 | - | 100 | - | 100 | - | 100 | 100 | - | 0 | <0.01 | 0.05 | 1 |
| 104 | 2.9 | 94 | 89 | - | 97 | - | 100 | - | 97 | 100 | - | 3 | <0.01 | 0.2 | 200 |
| 105 | 0.9 | 64 | 73 | - | 100 | - | 100 | - | 82 | 100 | - | 18 | <0.01 | 59.1 | 200 |
| 106 | 3 | 97 | 92 | - | 94 | - | 100 | - | 100 | 100 | - | 0 | <0.01 | 0.1 | 10 |
| 107 | 0.4 | 100 | 100 | - | 100 | - | 100 | - | 100 | 100 | - | 0 | <0.01 | <0.01 | 0.01 |
| 108 | 2.8 | 36 | 61 | - | 67 | - | 64 | - | 76 | 88 | - | 24 | 0.06 | >200 | >200 |
| 109 | 3.8 | 41 | 50 | - | 52 | - | 65 | - | 67 | 61 | - | 33 | 0.18 | >200 | >200 |
| 110 | 2 | 42 | 63 | - | 25 | - | 67 | - | 58 | 67 | - | - | - | - | - |
| 111 | 3.7 | 73 | 77 | - | 80 | - | 82 | - | 91 | 95 | - | 9 | <0.01 | >200 | >200 |
| 112 | 0.6 | 57 | 71 | - | 100 | - | 100 | - | 100 | 86 | - | 0 | 0.001 | 33.3 | >200 |
| 113 | 1.1 | 38 | 46 | - | 92 | - | 92 | - | 46 | 46 | - | - | - | - | - |
| 114 | 2.5 | 80 | 87 | - | 90 | - | 97 | - | 93 | 100 | - | 7 | 0.00006 | 12.7 | 200 |
| 115 | 1 | 67 | 100 | - | 100 | - | 100 | - | 100 | 100 | - | 0 | 0.007 | 0.035 | 0.1 |
| 116 | 3.5 | 79 | 93 | - | 100 | - | 100 | - | 100 | 100 | - | 0 | 0.001 | 0.107 | 1 |
| 117 | 1.1 | 62 | 100 | - | 100 | - | 100 | - | 100 | 100 | - | 0 | 0.008 | 0.039 | 0.1 |
| 118 | 1.1 | 85 | 85 | - | 85 | - | 85 | - | 100 | 92 | - | 0 | <0.01 | >200 | >200 |
| 119 | 3.7 | 80 | 93 | - | 95 | - | 100 | - | 98 | 100 | - | 2 | <0.01 | 0.61 | 200 |
| 120 | 1 | 83 | 83 | - | 100 | - | 100 | - | 100 | 100 | - | 0 | 0.001 | 0.27 | 1 |
| 121 | 0.4 | 40 | 60 | - | 60 | - | 100 | - | 100 | 100 | - | 0 | 0.049 | 16.4 | 10 |
| 122 | 2.7 | 91 | 97 | - | 100 | - | 100 | - | 100 | 100 | - | 0 | <0.01 | 0.031 | 1 |
| 123 | 2.3 | 89 | 96 | - | 96 | - | 100 | - | 100 | 100 | - | 0 | <0.01 | 0.091 | 10 |
| 124 | 1.1 | 46 | 85 | - | 85 | - | 100 | - | 100 | 100 | - | 0 | 0.011 | 2.02 | 10 |
| 125 | 0.6 | 29 | 71 | - | 100 | - | 100 | - | 100 | 100 | - | 0 | 0.03 | 0.46 | 1 |
| 126 | 4.3 | 67 | 94 | - | 98 | - | 100 | - | 100 | 100 | - | 0 | 0.003 | 0.18 | 10 |

Figure S1. Average values of oospore germination of the analyzed samples on the control medium (G_0_). Circles and asterisks represent mild and extreme outliers, respectively. Samples are identified by numbers.


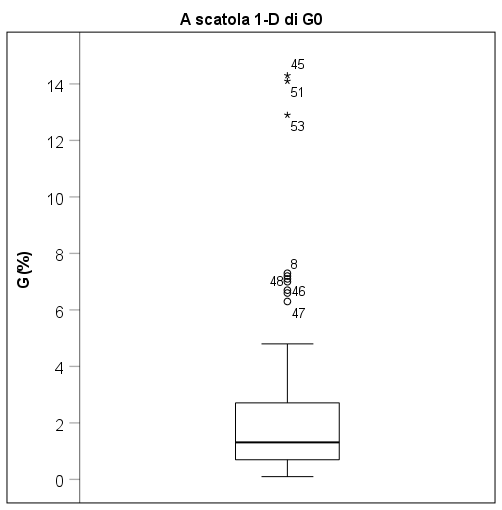


Table S3. Nucleotide sequences of the *β-tubulin* gene fragment of *P. viticola* strains obtained using the forward primer. Codon 239 is highlighted in yellow.

| Strain | Sequence |
| --- | --- |
| GirI3 | acagcTcGActGTCGTcGAgcCCTACAATGCCACGTTGTCGGTGCACCAGCTAGTTGAAAATGCTGACGAGGTCATGTGCCTGGACAATGAAGCCTTGTACGACATTTGCTTTCGTACTCTGAAACTCACCACTCCCACGTACGGCGACTTGAATCACTTGGTTTGTGCCGCCATGTCTGGCATCACCACA**TGC**CTTCGATTTCCCGGACAGCTGAATTCGGACTTACGAAAATTGGCTGTTAACCTGATTCCGTTCCCCCGTCTTCATTTCTTTATGATTGGATTTGCACCACTGACATCGCGCGGCTCGCAGCAGTATCGTGCGTTGACTGTACCTGAGTTAACCCAGCAGCAGTTTGATGCAAAAAATATGATGTGTGCCGCTGATCCTCGCCACGGCCGCTATTTAACAGCTGCGTGTATGTTCCGCGGACGTATGAGCACGAAGGAGGTTGACGAGCAGATGCTTAATGTTCAGAACAAAAACTCTTCGTACTTTGTTGAATGGATTCCCAACAATATCAAGGCTAGTGTGTGTGACATTCCGCCCAAGGGCCTGAAAATGAGCACCACGTTTATTGGTAACTCTACGGCTATTCAGGAAATGTTTAAGCGAGTATCCGAACAGTTTACAGCTATGTTCCGTCGTAAGGCTTTCTTGCACTGGTACACTGGTGAGttttgggaacaaaaaaa |
| GraI7 | agcgtctGACTGtCGTCGAgcCCTACAATGCCACGtTGTCGGTGCACCAGCTAGTTGAAAATGCTGACGAGGTCATGTGCCTGGACAATGAAGCCTTGTACGACATTTGCTTTCGTACTCTGAAACTCACCACTCCCACGTACGGCGACTTGAATCACTTGGTTTGTGCCGCCATGTCTGGCATCACCACA**TGC**CTTCGATTTCCCGGACAGCTGAATTCGGACTTACGAAAATTGGCTGTTAACCTGATTCCGTTCCCCCGTCTTCATTTCTTTATGATTGGATTTGCACCACTGACATCGCGCGGCTCGCAGCAGTATCGTGCGTTAACTGTACCTGAGTTAACCCAGCAGCAGTTTGATGCAAAAAATATGATGTGTGCCGCTGATCCTCGCCACGGCCGCTATTTAACAGCTGCGTGTATGTTCCGCGGACGTATGAGCACGAAGGAGGTTGACGAGCAGATGCTAAATGTTCAGAACAAAAACTCTTCGTACTTTGTTGAATGGATTCCCAACAATATCAAGGCTAGTGTGTGTGACATTCCGCCCAAGGGCCTGAAAATGAGCACCACGTTTATTGGTAACTCTACGGCTATTCAGGAAATGTTTAAGCGAGTATCCGAACAGTTTACAGCTATGTTCCGTCGTAAGGCTTTCTTGCACTGGTACACTGGTGAGGtAatgGGACGAAa |
| GheR4 | acaGttGACtGTCGTtGAgcCCTACAATGCCACGTTGTCGGTGCACCAGCTAGTTGAAAATGCTGACGAGGTCATGTGCCTGGACAATGAAGCCTTGTACGACATTTGCTTTCGTACTCTGAAACTCACCACTCCCACGTACGGCGACcTGAATCACTTGGTTTGTGCCGCCATGTCTGGCATCACCACA**RGC**CTTCGATTTCCCGGACAGCTGAATTCGGACTTACGAAAATTGGCTGTTAAtCTGATTCCGTTCCCCCGTCTTCATTTCTTTATGATTGGATTTGCACCAtTGACATCGCGCGGCTCGCAGCAGTATCGTGCGTTgACTGTACCTGAGTTAACCCAGCAGCAGTTTGATGCAAAAAATATGATGTGTGCCGCTGATCCTCGCCACGGCCGCTATTTAACAGCTGCGTGTATGTTCCGCGGACGTATGAGCACGAAGGAGGTTGACGAGCAGATGCTAAATGTTCAGAACAAAAACTCTTCGTACTTTGTTGAATGGATTCCCAACAATATCAAGGCTAGTGTGTGTGACATTCCGCCCAAGGGCCTGAAAATGAGCACCACGTTTATTGGTAACTCTACGGCTATTCAGGAAATGTTTAAGCGAGTATCCGAACAGTTTACAGCTATGTTcCGTCGTAAGGCTTTCTTGCACTGGTACACTGGTGAGggaatgggaacgaaaa |
| GheR5 | atcgTtgActGTCGTtcGAgcCCTACAaTGCCACGTTGTCGGTGCACCAGCTAGTTGAAAATGCTGACGAGGTCATGTGCCTGGACAATGAAGCCTTGTACGACATTTGCTTTCGTACTCTGAAACTCACCACTCCCACGTACGGCGACCTGAATCACTTGGTTTGTGCCGCCATGTCTGGCATCACCACA**AGC**CTTCGATTTCCCGGACAGCTGAATTCGGACTTACGAAAATTGGCTGTTAACCTGATTCCGTTCCCCCGTCTTCATTTCTTTATGATTGGATTTGCACCATTGACATCGCGCGGCTCGCAGCAGTATCGTGCGTTGACTGTACCTGAGTTAACCCAGCAGCAGTTTGATGCAAAAAATATGATGTGTGCCGCTGATCCTCGCCACGGCCGCTATTTAACAGCTGCGTGTATGTTCCGCGGACGTATGAGCACGAAGGAGGTTGACGAGCAGATGCTAAATGTTCAGAACAAAAACTCTTCGTACTTTGTTGAATGGATTCCCAACAATATCAAGGCTAGTGTGTGTGACATTCCGCCCAAGGGCCTGAAAATGAGCACCACGTTTATTGGTAACTCTACGGCTATTCAGGAAATGTTTAAGCGAGTATCCGAACAGTTTACAGCTATGTTCCGTCGTAAGGCTTTCTTGCACTGGTACACTGGTGAGgaaaggGGaaagAAAaaaa |
| GheR6 | aaagTcGactGTCGTtcGAgcCCTACAATGCCACGTTGTCGGTGCACCAGCTAGTTGAAAATGCTGACGAGGTCATGTGCCTGGACAATGAAGCCTTGTACGACATTTGCTTTCGTACTCTGAAACTCACCACTCCCACGTACGGCGACcTGAATCACTTGGTTTGTGCCGCCATGTCTGGCATCACCACA**AGC**CTTCGATTTCCCGGACAGCTGAATTCGGACTTACGAAAATTGGCTGTTAAtCTGATTCCGTTCCCCCGTCTTCATTTCTTTATGATTGGATTTGCACCAtTGACATCGCGCGGCTCGCAGCAGTATCGTGCGTTaACTGTACCTGAGTTAACCCAGCAGCAGTTTGATGCAAAAAATATGATGTGTGCCGCTGATCCTCGCCACGGCCGCTATTTAACAGCTGCGTGTATGTTCCGCGGACGTATGAGCACGAAGGAGGTTGACGAGCAGATGCTAAATGTTCAGAACAAAAACTCTTCGTACTTTGTTGAATGGATTCCCAACAATATCAAGGCTAGTGTGTGTGACATTCCGCCCAAGGGCCTGAAAATGAGCACCACGTTTATTGGTAACTCTACGGCTATTCAGGAAATGTTTAAGCGAGTATCCGAACAGTTTACAGCTATGTTcCGTCGTAAGGCTTTCTTGCACTGGTACACTGGTGaggttttggGaaagAAAaaaaaaaaaa |
| GheR9 | ggcgtcgactGTCGTcgAgcCCTACAATGCCACGTTGTCGGTGCACCAGCTAGTTGAAAATGCTGACGAGGTCATGTGCCTGGACAATGAAGCCTTGTACGACATTTGCTTTCGTACTCTGAAACTCACCACTCCCACGTACGGCGACcTGAATCACTTGGTTTGTGCCGCCATGTCTGGCATCACCACA**AGC**CTTCGATTTCCCGGACAGCTGAATTCGGACTTACGAAAATTGGCTGTTAAtCTGATTCCGTTCCCCCGTCTTCATTTCTTTATGATTGGATTTGCACCAtTGACATCGCGCGGCTCGCAGCAGTATCGTGCGTTGACTGTACCTGAGTTAACCCAGCAGCAGTTTGATGCAAAAAATATGATGTGTGCCGCTGATCCTCGCCACGGCCGCTATTTAACAGCTGCGTGTATGTTCCGCGGACGTATGAGCACGAAGGAGGTTGACGAGCAGATGCTAAATGTTCAGAACAAAAACTCTTCGTACTTTGTTGAATGGATTCCCAACAATATCAAGGCTAGTGTGTGTGACATTCCGCCCAAGGGCCTGAAAATGAGCACCACGTTTATTGGTAACTCTACGGCTATTCAGGAAATGTTTAAGCGAGTATCCGAACAGTTTACAGCTATGTTtCGTCGTAAGGCTTTCTTGCACTGGTACACTGGTGAGGTAattGGGACGAAa |
| MarR1 | aacgTtGActGTCGTTcGAgcCCTACAATGCCACGTTGTCGGTGCACCAGCTAGTTGAAAATGCTGACGAGGTCATGTGCCTGGACAATGAAGCCTTGTACGACATTTGCTTTCGTACTCTGAAACTCACCACTCCCACGTACGGCGACtTGAATCACTTGGTTTGTGCCGCCATGTCTGGCATCACCACA**RGC**CTTCGATTTCCCGGACAGCTGAATTCGGACTTACGAAAATTGGCTGTTAAcCTGATTCCGTTCCCCCGTCTTCATTTCTTTATGATTGGATTTGCACCAtTGACATCGCGCGGCTCGCAGCAGTATCGTGCGTTgACTGTACCTGAGTTAACCCAGCAGCAGTTTGATGCAAAAAATATGATGTGTGCCGCTGATCCTCGCCACGGCCGCTATTTAACAGCTGCGTGTATGTTCCGCGGACGTATGAGCACGAAGGAGGTTGACGAGCAGATGCTAAATGTTCAGAACAAAAACTCTTCGTACTTTGTTGAATGGATTCCCAACAATATCAAGGCTAGTGTGTGTGACATTCCGCCCAAGGGCCTGAAAATGAGCACCACGTTTATTGGTAACTCTACGGCTATTCAGGAAATGTTTAAGCGAGTATCCGAACAGTTTACAGCTATGTTcCGTCGTAAGGCTTTCTTGCACTGGTACACTGGTGAGtaatgGGaacga |
| MarR6 | aacgtcGActGTCGTtcGAgcCCTACAATGCCACGTTGTCGGTGCACCAGCTAGTTGAAAATGCTGACGAGGTCATGTGCCTGGACAATGAAGCCTTGTACGACATTTGCTTTCGTACTCTGAAACTCACCACTCCCACGTACGGCGACcTGAATCACTTGGTTTGTGCCGCCATGTCTGGCATCACCACA**RGC**CTTCGATTTCCCGGACAGCTGAATTCGGACTTACGAAAATTGGCTGTTAAcCTGATTCCGTTCCCCCGTCTTCATTTCTTTATGATTGGATTTGCACCAtTGACATCGCGCGGCTCGCAGCAGTATCGTGCGTTgACTGTACCTGAGTTAACCCAGCAGCAGTTTGATGCAAAAAATATGATGTGTGCCGCTGATCCTCGCCACGGCCGCTATTTAACAGCTGCGTGTATGTTCCGCGGACGTATGAGCACGAAGGAGGTTGACGAGCAGATGCTAAATGTTCAGAACAAAAACTCTTCGTACTTTGTTGAATGGATTCCCAACAATATCAAGGCTAGTGTGTGTGACATTCCGCCCAAGGGCCTGAAAATGAGCACCACGTTTATTGGTAACTCTACGGCTATTCAGGAAATGTTTAAGCGAGTATCCGAACAGTTTACAGCTATGTTcCGTCGTAAGGCTTTCTTGCACTGGTACACTGGTGAGtgtttggGaaagAAAaaaaaaaaa |
| MarR11 | accggggggggggggataagGcctaacaaTGCCACGTTGTCGGTGCACCAGCTAGTTGAAAATGCTGACGAGGTCATGTTGCCTGGACAATGAAGCCTTGTACGACATTTGCTTTCGTACTCTGAAACTCACCACTCCCACGTACGGCGACCTGAATCACTTGGTTTGTGCCGCCATGTCTGGCATCACCACA**WSC**CTTCGATTTCCCGGACAGCTGAATTCGGACTTACGAAAATTGGCTGTTAACCTGATTCCGTTCCCCCGTCTTCATTTCTTTATGATTGGATTTGCACCATTGACATCGCGCGGCTCGCAGCAGTATCGTGCGTTGACTGTACCTGAGTTAACCCAGCAGCAGTTTGATGCAAAAAATATGATGTGTGCCGCTGATCCTCGCCACGGCCGCTATTTAACAGCTGCGTGTATGTTCCGCGGACGTATGAGCACGAAGGAGGTTGACGAGCAGATGCTAAATGTTCAGAACAAAAACTCTTCGTACTTTGTTGAATGGATTCCCAACAATATCAAGGCTAGTGTGTGTGACATTCCGCCCAAGGGCCTGAAAATGAGCACCACGTTTATTGGTAACTCTACGGCTATTCAGGAAATGTTTAAGCGAGTATCCGAACAGTTTACAGCTATGTTTCGTCGTAAGGCTTTCTTGCACTGGTACACTGGTGAGGGTATGGACGAAat |
| CasTNT11 | acggtcgActGTCGTtcgagcCCTACAATGCCACGTTGTCGGTGCACCAGCTAGTTGAAAATGCTGACGAGGTCATGTGCCTGGACAATGAAGCCTTGTACGACATTTGCTTTCGTACTCTGAAACTCACCACTCCCACGTACGGCGACCTGAATCACTTGGTTTGTGCCGCCATGTCTGGCATCACCACA**KGC**CTTCGATTTCCCGGACAGCTGAATTCGGACTTACGAAAATTGGCTGTTAACCTGATTCCGTTCCCCCGTCTTCATTTCTTTATGATTGGATTTGCACCATTGACATCGCGCGGCTCGCAGCAGTATCGTGCGTTGACTGTACCTGAGTTAACCCAGCAGCAGTTTGATGCAAAAAATATGATGTGTGCCGCTGATCCTCGCCACGGCCGCTATTTAACAGCTGCGTGTATGTTCCGCGGACGTATGAGCACGAAGGAGGTTGACGAGCAGATGCTAAATGTTCAGAACAAAAACTCTTCGTACTTTGTTGAATGGATTCCCAACAATATCAAGGCTAGTGTGTGTGACATTCCGCCCAAGGGCCTGAAAATGAGCACCACGTTTATTGGTAACTCTACGGCTATTCAGGAAATGTTTAAGCGAGTATCCGAACAGTTTACAGCTATGTTCCGTCGTAAGGCTTTCTTGCACTGGTACACTGGTGAGtaatgGGaacaaAAaaaa |
| CasTNT17 | aacgttgACTGTCGtTGAacCCTACAATGCCACGTTGTCGGTGCACCAGCTAGTTGAAAATGCTGACGAGGTCATGTGCCTGGACAATGAAGCCTTGTACGACATTTGCTTTCGTACTCTGAAACTCACCACTCCCACGTACGGCGACCTGAATCACTTGGTTTGTGCCGCCATGTCTGGCATCACCACA**KGC**CTTCGATTTCCCGGACAGCTGAATTCGGACTTACGAAAATTGGCTGTTAATCTGATTCCGTTCCCCCGTCTTCATTTCTTTATGATTGGATTTGCACCATTGACATCGCGCGGCTCGCAGCAGTATCGTGCGTTGACTGTACCTGAGTTAACCCAGCAGCAGTTTGATGCAAAAAATATGATGTGTGCCGCTGATCCTCGCCACGGCCGCTATTTAACAGCTGCGTGTATGTTCCGCGGACGTATGAGCACGAAGGAGGTTGACGAGCAGATGCTAAATGTTCAGAACAAAAACTCTTCGTACTTTGTTGAATGGATTCCCAACAATATCAAGGCTAGTGTGTGTGACATTCCGCCCAAGGGCCTGAAAATGAGCACCACGTTTATTGGTAACTCTACGGCTATTCAGGAAATGTTTAAGCGAGTATCCGAACAGTTTACAGCTATGTTTCGTCGTAAGGCTTTCTTGCACTGGTACACTGGTGAGGTaAtGGACgaAaaa |
| CazR6 | cccggggggtggggaagaAaggcataAATGCCACGTTGTCGGTGCACCAGCTAGTTGAAAATGCTGACGAGGTCATGTTGCCTGGACAATGAAGCCTTGTACGACATTTGCTTTCGTACTCTGAAACTCACCACTCCCACGTACGGCGACTTGAATCACTTGGTTTGTGCCGCCATGTCTGGCATCACCACA**RGC**CTTCGATTTCCCGGACAGCTGAATTCGGACTTACGAAAATTGGCTGTTAATCTGATTCCGTTCCCCCGTCTTCATTTCTTTATGATTGGATTTGCACCATTGACATCGCGCGGCTCGCAGCAGTATCGTGCGTTGACTGTACCTGAGTTAACCCAGCAGCAGTTTGATGCAAAAAATATGATGTGTGCCGCTGATCCTCGCCACGGCCGCTATTTAACAGCTGCGTGTATGTTCCGCGGACGTATGAGCACGAAGGAGGTTGACGAGCAGATGCTAAATGTTCAGAACAAAAACTCTTCGTACTTTGTTGAATGGATTCCCAACAATATCAAGGCTAGTGTGTGTGACATTCCGCCCAAGGGCCTGAAAATGAGCACCACGTTTATTGGTAACTCTACGGCTATTCAGGAAATGTTTAAGCGAGTATCCGAACAGTTTACAGCTATGTTCCGTCGTAAGGCTTTCTTGCACTGGTACACTGGTGAGGGTAATGGACGAagaa |
| PasR1 | taagtcgactGTCGtTGAagcCCTACAATGCCACGTTGTCGGTGCACCAGCTAGTTGAAAATGCTGACGAGGTCATGTGCCTGGACAATGAAGCCTTGTACGACATTTGCTTTCGTACTCTGAAACTCACCACTCCCACGTACGGCGACCTGAATCACTTGGTTTGTGCCGCCATGTCTGGCATCACCACA**RGC**CTTCGATTTCCCGGACAGCTGAATTCGGACTTACGAAAATTGGCTGTTAATCTGATTCCGTTCCCCCGTCTTCATTTCTTTATGATTGGATTTGCACCATTGACATCGCGCGGCTCGCAGCAGTATCGTGCGTTGACTGTACCTGAGTTAACCCAGCAGCAGTTTGATGCAAAAAATATGATGTGTGCCGCTGATCCTCGCCACGGCCGCTATTTAACAGCTGCGTGTATGTTCCGCGGACGTATGAGCACGAAGGAGGTTGACGAGCAGATGCTAAATGTTCAGAACAAAAACTCTTCGTACTTTGTTGAATGGATTCCCAACAATATCAAGGCTAGTGTGTGTGACATTCCGCCCAAGGGCCTGAAAATGAGCACCACGTTTATTGGTAACTCTACGGCTATTCAGGAAATGTTTAAGCGAGTATCCGAACAGTTTACAGCTATGTTTCGTCGTAAGGCTTTCTTGCACTGGTACACTGGTGAGGtaattGgaACgAAaaa |
| PasR4 | ttaGTcgActGTCGTTGAgcCCTACAATGCCACGTTGTCGGTGCACCAGCTAGTTGAAAATGCTGACGAGGTCATGTGCCTGGACAATGAAGCCTTGTACGACATTTGCTTTCGTACTCTGAAACTCACCACTCCCACGTACGGCGACCTGAATCACTTGGTTTGTGCCGCCATGTCTGGCATCACCACA**RGC**CTTCGATTTCCCGGACAGCTGAATTCGGACTTACGAAAATTGGCTGTTAATCTGATTCCGTTCCCCCGTCTTCATTTCTTTATGATTGGATTTGCACCATTGACATCGCGCGGCTCGCAGCAGTATCGTGCGTTGACTGTACCTGAGTTAACCCAGCAGCAGTTTGATGCAAAAAATATGATGTGTGCCGCTGATCCTCGCCACGGCCGCTATTTAACAGCTGCGTGTATGTTCCGCGGACGTATGAGCACGAAGGAGGTTGACGAGCAGATGCTAAATGTTCAGAACAAAAACTCTTCGTACTTTGTTGAATGGATTCCCAACAATATCAAGGCTAGTGTGTGTGACATTCCGCCCAAGGGCCTGAAAATGAGCACCACGTTTATTGGTAACTCTACGGCTATTCAGGAAATGTTTAAGCGAGTATCCGAACAGTTTACAGCTATGTTTCGTCGTAAGGCTTTCTTGCACTGGTACACTGGTGAgtaaattGGAaCaAAAa |
| PasR9 | tcacgtcgactGTCGTTGAgcCCTACAATGCCACGTTGTCGGTGCACCAGCTAGTTGAAAATGCTGACGAGGTCATGTGCCTGGACAATGAAGCCTTGTACGACATTTGCTTTCGTACTCTGAAACTCACCACTCCCACGTACGGCGACCTGAATCACTTGGTTTGTGCCGCCATGTCTGGCATCACCACA**RGC**CTTCGATTTCCCGGACAGCTGAATTCGGACTTACGAAAATTGGCTGTTAATCTGATTCCGTTCCCCCGTCTTCATTTCTTTATGATTGGATTTGCACCATTGACATCGCGCGGCTCGCAGCAGTATCGTGCGTTGACTGTACCTGAGTTAACCCAGCAGCAGTTTGATGCAAAAAATATGATGTGTGCCGCTGATCCTCGCCACGGCCGCTATTTAACAGCTGCGTGTATGTTCCGCGGACGTATGAGCACGAAGGAGGTTGACGAGCAGATGCTAAATGTTCAGAACAAAAACTCTTCGTACTTTGTTGAATGGATTCCCAACAATATCAAGGCTAGTGTGTGTGACATTCCGCCCAAGGGCCTGAAAATGAGCACCACGTTTATTGGTAACTCTACGGCTATTCAGGAAATGTTTAAGCGAGTATCCGAACAGTTTACAGCTATGTTTCGTCGTAAGGCTTTCTTGCACTGGTACACTGGTGAGGtttggggagagAAAaaaaaa |
| PasR10 | cccgtGgatgtcggtagAaGcCCTACAATGCCACGTTGTCGGTGCACCAGCTAGTTGAAaTGCTGACGAGGTCATGTGCCTGGACAATGAAGCCTTGTACGACATTTGCTTTCGTACTCTGAAACTCACCACTCCCACGTACGGCGACCTGAATCACTTGGTTTGTGCCGCCATGTCTGGCATCACCACA**RGC**CTTCGATTTCCCGGACAGCTGAATTCGGACTTACGAAAATTGGCTGTTAATCTGATTCCGTTCCCCCGTCTTCATTTCTTTATGATTGGATTTGCACCATTGACATCGCGCGGCTCGCAGCAGTATCGTGCGTTGACTGTACCTGAGTTAACCCAGCAGCAGTTTGATGCAAAAAATATGATGTGTGCCGCTGATCCTCGCCACGGCCGCTATTTAACAGCTGCGTGTATGTTCCGCGGACGTATGAGCACGAAGGAGGTTGACGAGCAGATGCTAAATGTTCAGAACAAAAACTCTTCGTACTTTGTTGAATGGATTCCCAACAATATCAAGGCTAGTGTGTGTGACATTCCGCCCAAGGGCCTGAAAATGAGCACCACGTTTATTGGTAACTCTACGGCTATTCAGGAAATGTTTAAGCGAGTATCCGAACAGTTTACAGCTATGTTTCGTCGTAAGGCTTTCTTGCACTGGTACACTGGTGAGGGTAatgGACGAAAa |
| TesR2 | cCggggggaggggtgaaAAagcCATAcAATGCCACGTTGTCGGTGCACCAGCTAGTTGAAAATGCTGACGAGGTCAtGTTGCCTGGACAATGAAGCCTTGTACGACATTTGCTTTCGTACTCTGAAACTCACCACTCCCACGTACGGCGACCTGAATCACTTGGTTTGTGCCGCCATGTCTGGCATCACCACA**RGC**CTTCGATTTCCCGGACAGCTGAATTCGGACTTACGAAAATTGGCTGTTAATCTGATTCCGTTCCCCCGTCTTCATTTCTTTATGATTGGATTTGCACCATTGACATCGCGCGGCTCGCAGCAGTATCGTGCGTTGACTGTACCTGAGTTAACCCAGCAGCAGTTTGATGCAAAAAATATGATGTGTGCCGCTGATCCTCGCCACGGCCGCTATTTAACAGCTGCGTGTATGTTCCGCGGACGTATGAGCACGAAGGAGGTTGACGAGCAGATGCTAAATGTTCAGAACAAAAACTCTTCGTACTTTGTTGAATGGATTCCCAACAATATCAAGGCTAGTGTGTGTGACATTCCGCCCAAGGGCCTGAAAATGAGCACCACGTTTATTGGTAACTCTACGGCTATTCAGGAAATGTTTAAGCGAGTATCCGAACAGTTTACAGCTATGTTTCGTCGTAAGGCTTTCTTGCACTGGTACACTGGTGAGGGTATGGACGAAa |
| TesR6 | ttgatGTCgTTGAgcCCTAaCAATGCCACGTTGTCGGTGCACCAGCTAGTTGAAAATGCTGACGAGGTCATGTGCCTGGACAATGAAGCCTTGTACGACATTTGCTTTCGTACTCTGAAACTCACCACTCCCACGTACGGCGACCTGAATCACTTGGTTTGTGCCGCCATGTCTGGCATCACCACA**RGC**CTTCGATTTCCCGGACAGCTGAATTCGGACTTACGAAAATTGGCTGTTAATCTGATTCCGTTCCCCCGTCTTCATTTCTTTATGATTGGATTTGCACCATTGACATCGCGCGGCTCGCAGCAGTATCGTGCGTTGACTGTACCTGAGTTAACCCAGCAGCAGTTTGATGCAAAAAATATGATGTGTGCCGCTGATCCTCGCCACGGCCGCTATTTAACAGCTGCGTGTATGTTCCGCGGACGTATGAGCACGAAGGAGGTTGACGAGCAGATGCTAAATGTTCAGAACAAAAACTCTTCGTACTTTGTTGAATGGATTCCCAACAATATCAAGGCTAGTGTGTGTGACATTCCGCCCAAGGGCCTGAAAATGAGCACCACGTTTATTGGTAACTCTACGGCTATTCAGGAAATGTTTAAGCGAGTATCCGAACAGTTTACAGCTATGTTTCGTCGTAAGGCTTTCTTGCACTGGTACACTGGTGAGGTaaatGGACGAA |
| TesR9 | cgcgtcgaggGtacggattgaaGGaATAcaATGCCACGTTGTCGGTGCACCAGCTAGTTGAAaTGCTGACGAGGTCATGTGCCTGGACAATGAAGCCTTGTACGACATTTGCTTTCGTACTCTGAAACTCACCACTCCCACGTACGGCGACCTGAATCACTTGGTTTGTGCCGCCATGTCTGGCATCACCACA**RGC**CTTCGATTTCCCGGACAGCTGAATTCGGACTTACGAAAATTGGCTGTTAATCTGATTCCGTTCCCCCGTCTTCATTTCTTTATGATTGGATTTGCACCATTGACATCGCGCGGCTCGCAGCAGTATCGTGCGTTGACTGTACCTGAGTTAACCCAGCAGCAGTTTGATGCAAAAAATATGATGTGTGCCGCTGATCCTCGCCACGGCCGCTATTTAACAGCTGCGTGTATGTTCCGCGGACGTATGAGCACGAAGGAGGTTGACGAGCAGATGCTAAATGTTCAGAACAAAAACTCTTCGTACTTTGTTGAATGGATTCCCAACAATATCAAGGCTAGTGTGTGTGACATTCCGCCCAAGGGCCTGAAAATGAGCACCACGTTTATTGGTAACTCTACGGCTATTCAGGAAATGTTTAAGCGAGTATCCGAACAGTTTACAGCTATGTTTCGTCGTAAGGCTTTCTTGCACTGGTACACTGGTGAGGttAATGGACGAAAa |
| TesR12 | tatttgactGtcGTTGAacCCTaaCaATGCCACGTTGTCGGTGCACCAGCTAGTTGAAAATGCTGACGAGGTCATGTGCCTGGACAATGAAGCCTTGTACGACATTTGCTTTCGTACTCTGAAACTCACCACTCCCACGTACGGCGACCTGAATCACTTGGTTTGTGCCGCCATGTCTGGCATCACCACA**RGC**CTTCGATTTCCCGGACAGCTGAATTCGGACTTACGAAAATTGGCTGTTAATCTGATTCCGTTCCCCCGTCTTCATTTCTTTATGATTGGATTTGCACCATTGACATCGCGCGGCTCGCAGCAGTATCGTGCGTTGACTGTACCTGAGTTAACCCAGCAGCAGTTTGATGCAAAAAATATGATGTGTGCCGCTGATCCTCGCCACGGCCGCTATTTAACAGCTGCGTGTATGTTCCGCGGACGTATGAGCACGAAGGAGGTTGACGAGCAGATGCTAAATGTTCAGAACAAAAACTCTTCGTACTTTGTTGAATGGATTCCCAACAATATCAAGGCTAGTGTGTGTGACATTCCGCCCAAGGGCCTGAAAATGAGCACCACGTTTATTGGTAACTCTACGGCTATTCAGGAAATGTTTAAGCGAGTATCCGAACAGTTTACAGCTATGTTTCGTCGTAAGGCTTTCTTGCACTGGTACACTGGTGAGGtaattgGGacGaAA |
